# Therapiestrategien von repetitiven Vokalisationen bei Demenz: Ein systematisches Review

**DOI:** 10.1007/s40211-024-00511-5

**Published:** 2024-10-15

**Authors:** Samuel Taubenheim, Arnim Quante

**Affiliations:** 1 Klinik für Psychiatrie und Psychotherapie, Charité – Universitätsmedizin Berlin, Berlin, Deutschland; 2 Klinik für Psychiatrie, Psychotherapie und Psychosomatik, Friedrich von Bodelschwingh-Klinik, Landhausstraße 33–35, 10717 Berlin, Deutschland

**Keywords:** Schlaf, Agitation, Verhaltensstörungen, Behandlungsansätze, Rufen, Sleep, Agitation, Behavioral and psychological symptoms of dementia, Treatment approaches, Vocalization

## Abstract

**Hintergrund:**

Vokalisationen bei Demenzerkrankten sind sich wiederholende, verbale Äußerungen, die aufgrund ihrer Lautstärke, Häufigkeit und/oder sozialer Unangemessenheit zu negativen Auswirkungen auf Erkrankte und weitere anwesende Individuen führen. Die Prävalenz liegt bei bis zu 30 %. Aufgrund einer stark zunehmenden Inzidenz weltweiter Demenzerkrankungen und einer geringen Anzahl randomisierter kontrollierter Studien hinsichtlich der Behandlung repetitiver Vokalisationen, soll dieses systematische Review eine Zusammenfassung existierender Arbeiten über die Effektivität möglicher pharmakotherapeutischer und nicht pharmakologischer Therapien geben.

**Methoden:**

Das systematische Review basiert auf den PRISMA-Richtlinien und die Anmeldung erfolgte auf der Registrierungsseite PROSPERO (Registriernummer: CRD42023486344). Die Literaturrecherche fand in den Datenbanken PubMed und Embase statt. Aufgrund einer mangelnden Datenlage wurden ebenfalls Fallberichte eingeschlossen.

**Ergebnisse:**

Von 2635 relevanten Artikeln wurden 25 Arbeiten in das Review inkludiert. Es konnte für SSRI, Pregabalin, Gabapentin und für die Antipsychotika Haloperidol und Risperidon anhand von wenigen Fallberichten eine Wirksamkeit belegt werden, die allerdings mit Nebenwirkungen behaftet war. Nicht pharmakologische Interventionen zeigten sich ebenfalls wirksam.

**Schlussfolgerung:**

Medikamentöse und nichtmedikamentöse Interventionen sind wirksame Behandlungsansätze hinsichtlich repetitiver Vokalisationen bei Demenzerkrankten. Aufgrund der Tatsache, dass kaum randomisiert-kontrollierte Studien vorliegen, müssen die Ergebnisse dieses systematischen Reviews mit Vorsicht interpretiert werden. Die Ergebnisse dieses Reviews zeigen, dass bei vielen Interventionen randomisiert-kontrollierte Studien zu fordern sind.

**Zusatzmaterial online:**

Zusätzliche Informationen sind in der Online-Version dieses Artikels (10.1007/s40211-024-00511-5) enthalten.

## Abstract (en)

**Background:**

Vocalizations in dementia patients are repetitive verbal expressions that, due to their volume, frequency, and/or social inappropriateness, cause negative effects on patients and other individuals present. The prevalence ranges up to 30%. Given the increasing global incidence of dementia and the limited number of randomized controlled trials about treating repetitive vocalizations, this systematic review provides a summary of existing works on the effectiveness of potential pharmacotherapeutic and nonpharmacological therapies.

**Methods:**

The systematic review follows the Preferred Reporting Items for Systematic Reviews and Meta-Analyses (PRISMA) guidelines and was registered on the International Prospective Register of Systematic Reviews (PROSPERO) registration page (registration number: CRD42023486344). Literature search was conducted in PubMed and Embase databases. Due to insufficient data, case reports were also included.

**Results:**

Of 2635 articles, 25 studies were included in the review. Efficacy was demonstrated for selective serotonin reuptake inhibitors (SSRIs), pregabalin, gabapentin, and the antipsychotics haloperidol and risperidone based on a few case reports, albeit with associated side effects. Nonpharmacological interventions also showed efficacy.

**Conclusion:**

Both pharmacological and nonpharmacological interventions are effective treatment approaches for repetitive vocalizations in dementia patients. Due to the fact that there are hardly any randomized controlled studies available, the results of this systematic review must be interpreted with caution. The results of this review show that randomized controlled trials are required for many interventions.

## Einleitung

Die Demenz ist ein Syndrom als Folge einer meist chronischen oder fortschreitenden Krankheit des Gehirns mit Störung vieler höherer kortikaler Funktionen, einschließlich Gedächtnis, Denken, Orientierung, Auffassung, Rechnen, Lernfähigkeit, Sprache und Urteilsvermögen [1]. Laut World Health Organisation leiden aktuell mehr als 55 Mio. Menschen weltweit an Demenz mit einer jährlichen Zunahme von etwa 10 Mio. Fällen [2]. Aktuell kann davon ausgegangen werden, dass die Gesamtzahl auf 78 Mio. Erkrankte im Jahr 2030 und auf 139 Mio. im Jahr 2050 ansteigen wird [3]. Eine häufige Erscheinung, die im Zusammenhang mit Demenz auftritt, sind repetitive Vokalisationen. Es handelt sich dabei um „laute und […] sich wiederholende verbale Äußerungen, die eine nachteilige Auswirkung auf anwesende Individuen aufgrund der Lautstärke, Häufigkeit und/oder sozialer Unangemessenheit haben [4]. Die Arten verbaler Ausdrücke eingeschlossen in der Definition […] beinhalten Schreie, Stöhnen, einzelne Wörter oder Sätze, wiederholte Fragen oder Nachfrage nach Aufmerksamkeit, Fluchen oder verbale Beschimpfung […]“ [4]. Die Prävalenz repetitiver Vokalisationen bei Demenz wird etwa auf 10–30 % geschätzt [4–7]. Besonders relevant ist, dass Vokalisationen über mehrere Monate, ohne erkennbare Ursache und bis zur körperlichen Erschöpfung bestehen können [8]. Diese Umstände können dazu führen, dass sich andere Bewohner:innen massiv gestört fühlen und diese ein erhöhtes Risiko für die Entwicklung von Ängsten und Agitationen aufweisen [9]. In anderen Studien wurde postuliert, dass Pflegekräfte, die regelmäßig bei Menschen mit repetitiven Vokalisationen eingesetzt werden, ein erhöhtes Risiko für Fehlzeiten aufweisen und zu einer „Burnout“-Symptomatik neigen [9]. Eine mögliche Konsequenz könnte nach persönlicher klinischer Erfahrung sogar die Kündigung des Heimplatzes sein, jedoch existieren hierzu keine validen Daten. Eine Kündigung ist dann möglich, wenn eine betroffene Person andere Bewohner:innen permanent stört [10]. Dies wiederum kann zu sozialer Isolation, Übermedikation und Vernachlässigungen führen [11]. Hinsichtlich der Therapie repetitiver Vokalisationen werden verschiedene Therapieansätze verfolgt, wobei medikamentöse und nichtmedikamentöse Behandlungsoptionen zur Verfügung stehen. Medikamentöse Therapien sind jedoch oft nebenwirkungsbehaftet [12].

Der Pathomechanismus für das Auftreten von Vokalisationen bei Demenz ist unklar. Es werden neuroanatomische Korrelate, die auf einer Schädigung oder Verletzung orbitofrontaler Strukturen basieren und mit einer Enthemmung einhergehen können, diskutiert [13]. Auch dorsolaterale präfrontale Strukturen, die zu Defiziten bei der Entscheidungsfindung führen können, werden hypothetisiert [13]. Bei neurodegenerativen Erkrankungen, aber auch bei (sub)akuten Ereignissen, können diese Strukturen geschädigt sein [13]. So konnte bei einer in dieser Arbeit erwähnten Patientin ein subakuter Hirninfarkt im Bereich des linken Gyrus rectus/medialer orbitofrontaler Gyrus (Brodmann-Gebiete 11–12) als mögliche Ursache identifiziert werden [13]. Der orbitofrontale Gyrus ist mit sensorischer Integration, Affekt, Entscheidungsfindung und Hedonismus verbunden [13]. Läsionen in dieser Region können zu kognitiven, Verhaltens- und emotionalen Symptomen führen [13].

Dementsprechend kann auch ein Zusammenhang zu möglichen Wirkungsweisen medikamentöser Therapien hergestellt werden. Vokalisationen werden als Teil von agitiertem Verhalten bei Personen mit Demenz eingeordnet. Medikamentöse Ansätze, die bei Agitation bereits Wirkung zeigten, könnten also auch bei Vokalisationen funktionieren. Unter anderem werden Defizite der cholinergen Neurotransmission, eine erhöhte D_2_/D_3_-Rezeptor-Verfügbarkeit und Defekte in der monoaminergen (5HT_2_-Rezeptor) Transmission diskutiert [14]. Dementsprechend stellen Acetylcholinesteraseinhibitoren, SSRIs, atypische Antipsychotika, Antiepileptika und stimmungsstabilisierende Substanzen medikamentöse Behandlungsoptionen dar [14].

Generell muss die Nutzung pharmakotherapeutischer Verfahren vor dem Hintergrund einer unklaren Studienlage hinsichtlich deren Wirksamkeit bei repetitiven Vokalisationen betrachtet werden. Um Nebenwirkungen zu reduzieren und eine erhöhte Wirksamkeit zu erreichen, gibt es verschiedene Überlegungen bezüglich nichtmedikamentöser Behandlungsoptionen wie die Musiktherapie, Massage, Lichttherapie oder die Elektrokonvulsionstherapie. Ein Review aus dem Jahr 2021 über nicht pharmakologische Interventionen bei disruptiven Vokalisationen schlussfolgert, dass Badetherapie zur Reduktion führen kann, Massagen jedoch eher heterogene Ergebnisse hervorbringen [15]. Die Studienlage über Interventionen bei repetitiven Vokalisationen ist sehr uneinheitlich. Es existiert nur eine geringe Auswahl systematischer Literatur und placebokontrollierter Studien. Dieses Review soll einen Überblick über pharmakologische und nichtpharmakologische Therapieansätze liefern.

## Methodik

Das systematische Review wurde vorab auf der Registrierungsseite PROSPERO angemeldet (Registriernummer: CRD42023486344). Es basiert auf den PRISMA-Richtlinien für systematische Reviews, wobei die Literaturrecherche in den medizinischen Datenbanken PubMed und Embase erfolgte.

Die ausgewählten Arbeiten wurden auf Basis von im Voraus getroffenen Auswahlkriterien in das Review eingeschlossen. Zunächst beschränkte sich der Veröffentlichungszeitraum der Publikationen auf die Zeitspanne zwischen 1990 und 2023. Publikationen mussten in deutscher und englischer Sprache verfasst sein. Hinsichtlich der Proband:innen war eine diagnostizierte Demenzerkrankung erforderlich. Dabei wurden alle Formen von Demenzerkrankungen inkludiert. Vokalisationen, beispielsweise bei Tic-Störungen oder Erkrankungen, die nicht mit einer Demenz assoziiert sind, wurden ausgeschlossen. Es mussten medikamentöse oder nichtmedikamentöse Interventionen zum Einsatz gekommen sein. Folgende Studiendesigns wurden inkludiert: placebokontrollierte Studien, Interventionsstudien, Beobachtungsstudien, Fallserien und Fallberichte. Es fand kein Einschluss von Studien ohne expliziten Bezug zu repetitiven Vokalisationen statt.

Die Literaturrecherche erfolgte unabhängig voneinander durch zwei Autoren im Zeitraum von November 2023 bis zum 22. April 2024. Folgende Suchbegriffe wurden konsentiert und verwendet: „Dementia“, „Vocalisation“, „Repetitive Vocalisation“, „Verbal Agitation“, „Screaming“, „Shouting“, „Vocally disruptive Behaviour“, „Noise Making“, „Verbal Agression“, „Yelling“, „Repetitious Mannerisms“ und „Persistent Vocalization“. Es erfolgte eine Kombination mit weiteren Suchbegriffen hinsichtlich der Therapieoptionen, unter anderem: „Antidepressants“, „Antipsychotics“, „Mood Stabilizers“, „Benzodiazepines“, „Anxiolytics“, „Stimulants“, „Anti-dementia Drugs“, „Acetylcholinesterase Inhibitors“, „Donepezil“, „ECT“, „Bathing“, „Massage“, „Aroma Therapy“, „Music“. Eine detaillierte tabellarische Darstellung der Suchstrategie mit Ergebnisanzahl ist im Anhang beigefügt worden.

Von besonderer Bedeutung bei der Datenextraktion waren vorrangig der *Cohen-Mansfield Agitation Inventory* (CMAI) und die *Pittsburgh Agitation Scale* (PAS) zur Objektivierung von möglichen Veränderungen der Intensität und Häufigkeit von Vokalisationen. Aufgrund der häufigen Verwendung des CMAI und davon meist der Unterkategorie „verbale nicht aggressive Subskala“ (VNAB) inklusive repetitiver Vokalisationen, sei kurz der Punktwert von 1–7 Punkten erläutert: 1 bedeutet kein Auftreten des Verhaltens, 7 Punkte werden bei mehrmalig stündlich auftretenden Vokalisationen über einen Zeitraum von 2 Wochen vergeben. Bezüglich der Pittsburgh Agitation Scale können über einen selbst festgelegten Bewertungszeitraum 0 Punkte, wenn keine aberranten Vokalisationen auftreten und 4 Punkte, wenn diese sehr laut und disruptiv sind, vergeben werden.

Zur Qualitätsbewertung der Studien wurde der Jadad-Score berechnet und aufgrund der hohen Anzahl an Fallberichten und -serien auch die STROBE-Checkliste angewendet. Der Jadad-Score bewertet vorliegende Studien anhand einer sachgerecht durchgeführten Randomisierung, Verblindung und der Begründung möglicher Drop-outs von Proband:innen. Die Skala reicht von −2 bis +5 Punkten. Studien, die mit einer Punktzahl von über 3 Punkten bewertet werden, gelten als qualitativ hochwertig, während Studien, die weniger als 3 Punkte erhalten, von niedriger Qualität sind. Der Jadad-Score weist jedoch Schwächen bei der Beurteilung nicht randomisierter Studien und Fallberichte auf, weshalb als weiteres Instrument die STROBE-Checkliste angewendet wurde. Die inkludierten Studien wurden dabei anhand von verschiedenen Items eingestuft. Dabei sind maximal 22 Punkte erfüllbar, die darüber Auskunft geben, ob Elemente wie eine ausführliche Auflistung der Proband:innencharakteristika angegeben sind.

## Ergebnisse

**Abb. 1:**
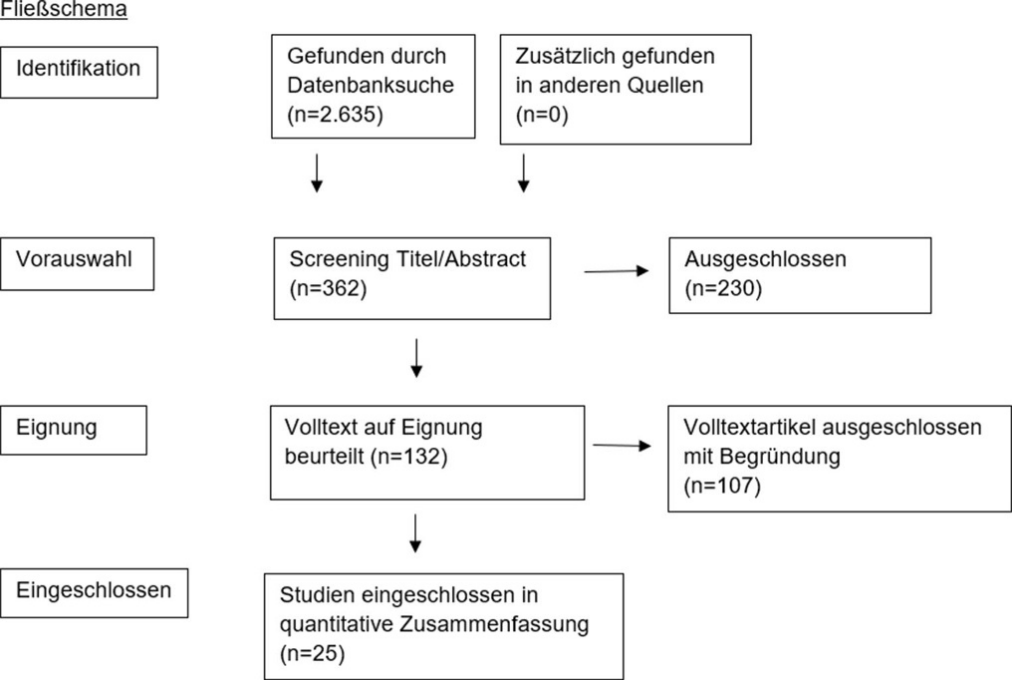
Fließschema der Studienrecherche nach den PRISMA-Kriterien

Der Einschluss vorausgewählter Studien erfolgte anhand eingangs erwähnter Kriterien. Eine genaue Auflistung der Vorgehensweise inklusive Vorauswahl und Begründung des Einschlusses für das systematische Review wurde im Anhang beigefügt. Die Vorauswahl der Studien sowie der Studieneinschluss ist im folgenden Fließschema grafisch dargestellt (Abb. 1). Insgesamt beinhaltet das hier vorliegende systematische Review fünfundzwanzig wissenschaftliche Arbeiten. Die Zusammenfassung der untersuchten Artikel erfolgte tabellarisch geordnet nach Studie, Erstautor:in und Veröffentlichungsjahr, Anzahl der Studienteilnehmer:innen mit Geschlechtsverteilung und Durchschnittsalter, Studiendesign, Messinstrument, Intervention, Ergebnis, Limitationen und möglichen Nebenwirkungen.

Im Folgenden werden einzelne pharmakologische Interventionen – unterteilt nach Substanzklassen – und nichtpharmakologische Interventionen vorgestellt und in der Tabelle im Zusatzmaterial online ausführlich aufgeführt.

## Pharmakologische Interventionen

### Antidepressiva

In einem Fallbericht mit zwei Proband:innen profitierten beide innerhalb der ersten 10 Tage von der Behandlung mit Citalopram in einer Dosis von zunächst 20 mg und später 40 mg am Tag [16]. Jedoch kehrte die Symptomatik nach Absetzen der Medikamente rasch wieder zurück [16]. Die Therapie mit Paroxetin führte in einer offenen Interventionsstudie mit 15 Proband:innen nach 3 Monaten zu einer Reduktion der Vokalisationen um 67–71 %, jedoch traten Nebenwirkungen wie die Verschlechterung eines Parkinsontremors und Gewichtszunahme auf [17].

### Antipsychotika

Risperidon erwies sich in einer Fallserie wirksam: Die Vokalisationen konnten auf weniger als 10 % des ursprünglichen Wertes reduziert werden, wobei auch hier die Symptomatik nach Absetzen wiederkehrte [12]. Haloperidol war Risperidon in einer Post-hoc-Analyse aus einer randomisierten, doppelblinden Studie nicht signifikant überlegen und ging stärker mit Nebenwirkungen wie Schlaflosigkeit und Somnolenz einher [18]. Die Antipsychotika Thioridazin, Haloperidol und Perphenazin brachten in einer weiteren Fallserie eine uneinheitliche Wirkung hinsichtlich repetitiver Vokalisationen hervor [19]. Dabei wurden durch den Einsatz von Thioridazin keine Veränderungen oder Verschlechterungen erzielt, hingegen konnte bei Anwendung von Haloperidol das gesamte Spektrum von Verbesserungen, ausbleibenden Effekten und Verschlechterungen beobachtet werden [19]. Perphenazin wurde lediglich bei einer Probandin eingesetzt [19]. Die Autoren haben einerseits diskrete Verbesserungen der Vokalisationen beobachtet, gleichzeitig aber auch Verschlechterungen, die jedoch nicht näher beschrieben wurden [19].

### Antikonvulsiva

Der Einsatz des Antikonvulsivums Gabapentin konnte in einem Fallbericht die Frequenz und Intensität von vokal-disruptivem Verhalten reduzieren, jedoch verbunden mit einem nebenwirkungsbedingten Therapieabbruch, da es zum Auftreten von Myoklonien kam [5]. Ein Fallbericht verdeutlichte, dass Pregabalin zu einer Reduktion von Intensität und Frequenz der Vokalisationen über einen Zeitraum von über 9 Monaten führte [5].

### Antidementiva/Acetylcholinesteraseinhibitoren

In der Sekundäranalyse einer Interventionsstudie mit 46 Proband:innen, die über 52 Wochen Donepezil erhalten hatten, konnten Verbesserungen bei 15 Proband:innen nach 3 Monaten und bei 12 Proband:innen nach 6 Monaten herausgestellt werden [20]. Generell kann durch den Einsatz von Acetylcholinesteraseinhibitoren und Memantine auf Verhaltensstörungen wie Agitation und Aggression, wenn auch uneinheitlich, eine leichte Wirksamkeit erzielt werden [21].

### Weitere pharmakologische Ansätze

Cannabis-Öl erwies sich in einem Fallbericht ebenfalls wirksam: Nach Versuchsdurchführung reduzierte sich der CMAI für verbal nicht aggressives Verhalten von durchschnittlich 4 auf 2 Punkte [22].

Zu vielen pharmakologischen Interventionen wurden keine Studien oder Fallberichte detektiert, obwohl diese durchaus einen Einfluss auf Vokalisationen haben könnten [23]. Insbesondere Psychostimulanzien und Anxiolytika wurden in dieser Indikation nicht recherchiert. Auch könnten in Zukunft Psychedelika in dieser Indikation auf eine mögliche Wirksamkeit überprüft werden.

### Nichtpharmakologische Interventionen

In einer Interventionsstudie konnte der Einsatz von schweren Gewichtsdecken, mit denen die Proband:innen während der Intervention bedeckt wurden, die Dauer der persistierenden Vokalisationen am Ende der Intervention signifikant reduzieren [11]. Die durchschnittliche Gesamtlänge der Vokalisationen bei Baseline-Bedingungen betrug 3,62 min (SD = 3,58) in einem 60-Minuten-Intervall, mit aufgelegten Decken 1,8 min (SD = 1,85) und nach Abnahme der Decken 0,9 min (SD = 0,99). Durch die Intervention konnte eine nicht signifikante Reduktion mit aufgelegter Decke (*p* = 0,0691) sowie eine signifikant geringere Dauer von persistierenden Vokalisation nach Entfernung der Decken (*p* = 0,033) erzielt werden [11].

Ebenfalls wurde in einer Interventionsstudie der Effekt von Audiotapes, welche die Geräusche eines Stroms oder Ozeans imitierten und den Proband:innen über Kopfhörer vorgespielt wurden, auf repetitive Vokalisationen überprüft [24]. Insgesamt konnte eine Reduktion verbaler Agitation um 11 % bei 9 von 13 auf die Intervention ansprechende Proband:innen erreicht werden [24].

Gruppenmusik bewirkte in einer weiteren Interventionsstudie eine statistisch signifikante Abnahme von verbal nicht aggressivem Verhalten nach 6 (*p* = 0,042) und 12 Sitzungen im Vergleich zum Vortest (*p* = 0,10) [25].

Weiterhin wurde eine Reduktion repetitiver Sätze von 60,8 % auf 27 % nach 4 Wochen durch personalisierte Musik erzielt [26].

Sensorische Stimulation mit der Hand reduzierte die vorliegenden Vokalisationen um 48 % während der Behandlung, jedoch verbunden mit einer erneuten Zunahme nach Behandlungsende [27].

Eine Longitudinalstudie, bei der eine sensorische Stimulation der Proband:innen erfolgte, stellte eine signifikante Abnahme von verbal agitiertem Verhalten in der Interventionsgruppe heraus [28]. Der CMAI für verbal agitiertes Verhalten der Interventionsgruppe sank von 14 Punkten im Pre-Trial auf 7 Punkte im Post-Trial und stieg im Follow-up auf 9 Punkte an [28]. Die Kontrollgruppe verzeichnete nach einer kurzen Reduktion von 12 auf 9 Punkte im Mid-Trial einen erneuten Anstieg auf 12 Punkte im Follow-up [28].

In einer randomisierten kontrollierten Studie erwies sich die sensorische Stimulation bei verbal agitiertem Verhalten im Vergleich zur Kontrollgruppe als effektiv [29]. Es wurden zwischen der Interventions- und Kontrollgruppe signifikante Effekte für den CMAI zwischen Woche 0 und Woche 16 (F2,36 = 3,460, *p* = 0,042, η^2^ = 0,155) erzielt [29]. Für die Interventions- und Kontrollgruppe ergaben sich entsprechende Punktwertveränderungen bezüglich des CMAI. Für die Interventionsgruppe wurden im Pre-Trial 11 Punkte, im Post-Trial 6 Punkte und im Follow-up 14 Punkte notiert [29]. Für die Kontrollgruppe ergab sich ein Wert von 9 Punkten im Pre-Trial; 12 Punkten im Mid-Trial und 9 Punkten im Post-Trial [29].

Hinsichtlich des Einsatzes topischer Cremes konnten folgende Effekte erzielt werden: in der Pittsburgh Agitation Scale (PAS) für aberrante Vokalisationen zeigte sich ein signifikanter Gruppeneffekt (*p* = 0,018) und eine statistisch signifikante Reduktion in der Interventionsgruppe (*p* = 0,020) in Woche 4 im Vergleich zur Kontrollgruppe [30]. Jedoch konnten diese signifikanten Effekte in Woche 8 und 10 nicht mehr dargestellt werden [30]. Der CMAI brachte einen statistisch signifikanten Gruppeneffekt (*p* = 0,012) und Zeit-Gruppen-Interaktionseffekt (*p* = 0,024) hervor [30]. Für die Interventionsgruppe (*p* = 0,007) konnten ebenfalls signifikante Veränderungen in Woche 4 im Vergleich zur Kontrolle beobachtet werden [30].

Die Durchführung einer Aromatherapie konnte in einer randomisierten kontrollierten Studie eine signifikante Veränderung des CMAI für verbal agitiertes nicht aggressives Verhalten nach 4 Wochen (Effekt −2,92 (0,91); T‑Wert = −3,22; *p*-Wert = 0,018) hervorbringen [31].

Durch Ausführung eines professionellen Schulungsprogramms ergaben sich für die Interventionsgruppe nach 3 Wochen signifikante Verbesserungen der Vokalisationen: Durchschnitt 18,71 (*p* = 0,008); Zweiter Nachtest nach 3 Monaten: 17,50, *p* = 0,010 [32]. Im Vergleich dazu die Kontrollgruppe: erster Nachtest 17,21, *p* = 0,014; zweiter Nachtest 17,63, *p* = 0,067 [32]. Es ergaben sich keine signifikanten Unterschiede im zweiten Nachtest der Kontrollgruppe [32]. Es wurden keine expliziten Punktwertveränderungen für den CMAI angegeben, sondern für verbal nicht aggressives Verhalten (VNAB) [32]. Für den VNAB inklusive repetitiver Vokalisationen zeigt sich in der Interventionsgruppe eine Abnahme des Gesamtdurchschnitts zwischen dem 1. und 2. Nachtest (von 18,71 auf 17,5 Punkte), während sich in der Kontrollgruppe eine Zunahme des Gesamtdurchschnitts zwischen dem 1. und 2. Nachtest darstellt (von 17,21 auf 17,63 Punkte) [32].

Die Anwendung eines weiteren Schulungsprogramms konnte folgende Ergebnisse erzielen: Zwischen Baseline und Woche 8 kam es zu einer signifikanten Reduktion für verbal-agitiert nicht aggressives Verhalten in der Interventionsgruppe von -0,41, (*p* < 0,001), jedoch nicht in der Kontrollgruppe (−0,04) [33]. Zwischen Baseline und Woche 20 ergab sich eine signifikante Reduktion von −0,47 (*p* < 0,001) in der Interventionsgruppe, nicht jedoch in der Kontrollgruppe (−0,03) [30]. In der Follow-up-Untersuchung waren die Effekte weiterhin stabil (Interventionsgruppe *p* < 0,0001; Kontrollgruppe *p* = 0,832) [33].

In einem Fallbericht erwies sich eine tiergestützte Therapie, bei der eine Probandin Zeit mit der Pflege eines Hundes verbrachte, als sehr effektiv [34]. Allerdings traten nach der Intervention andere unerwünschte, nachteilige Verhaltensweisen wie das Greifen nach Personen auf [34].

Lichttherapie führte in einer randomisierten kontrollierten Studie mit 60 Proband:innen zu einer signifikanten Veränderung von verbal agitiertem nicht aggressiven Verhalten direkt nach Beendigung der Intervention (β = −0,63, Waldχ2 = 13,39, *p* < 0,001), jedoch wurden keine expliziten Punktwertveränderungen detektiert [35].

Schließlich konnten Fallberichte zur Effektivität der Elektrokonvulsionstherapie (EKT) ein völliges Einstellen der Vokalisationen nach der dritten Behandlung darstellen, wobei dieser Effekt auch 4 Wochen nach der Entlassung anhielt [8].

Eine Reduktion vokal-disruptiven Verhaltens nach der dritten EKT-Behandlung wurde ebenfalls in einem Fallbericht beschrieben [36].

EKT führte zu einer Verbesserung von aberranten Vokalisationen, gemessen mittels PAS, von 3 Punkten auf 1 Punkt [37]. Es traten jedoch reversible Nebenwirkungen wie Verwirrtheit oder zunehmende Gangstörungen auf [37].

Folgend sind alle Ergebnisse sowie die Qualitätsbewertung der Studien mittels Jadad-Score und STROBE-Checkliste dargestellt (Tab. 1).

**Tab. 1:** Jadad-Score und STROBE-Checkliste sowie Effekte, Wirksamkeitsdauer und Nebenwirkungen der verschiedenen inkludierten Arbeiten

Intervention	Jadad-Score für Studiendesign (von −2 bis +5)	Verbesserung (+) Kein Effekt (0) Verschlechterung (−)	Dauer der Wirksamkeit	Relevante Nebenwirkungen	STROBE-Checkliste (wenn zutreffend Anzahl erfüllter Items max. 22)	
Antidepressivum Citalopram [16] *n* =2 Endpunkt: Verhaltensänderung	−2	+	A: 10 Tage, Rezidiv 5 Tage nach Therapieende B: 10 Tage	k.A.	−	
Antidepressivum Paroxetin [17] *n* = 15 Endpunkt: CMAI	−2	+	3 Monate	Verschlechterung Parkinsontremor, Diarrhö, Gewichtszunahme	13	
Antipsychotikum Risperidon [12] *n* =2 Endpunkt: Vokalisationsfrequenz	−2	+	A: 3 Wochen B: 8 Wochen	k.A.	–	
Antipsychotikum Risperidon [18]	5	+	8 Wochen	Schlaflosigkeit, Somnolenz, Zunahme extrapyramidaler Symptomatik	17	
Antipsychotikum Haloperidol [18] *n* = 120 Endpunkt: CMAI‑K	k.A.	0	8 Wochen	k.A.	k.A.	
Antipsychotika Thioridazin, Haloperidol und Perphenazin [19] *n* = 11 Endpunkt: Verhaltensänderung	−1	+/0/−	–	k.A.	–	
Antiepileptikum Gabapentin [5]	−2	+	−	Myoklonisches Zucken	–	
Antiepileptikum Pregabalin [5] *n* =1 Endpunkt: Verhaltensänderung	−2	+	9 Monate	k.A.	k.A.	
Acetylcholinesteraseinhibitor Donepezil [20] *n* = 46 Endpunkt: Goal Attainment Scaling	−2	+	Bis zu 12 Monate	k.A.	17	
Donepezil/Memantin [21] Endpunkt: NPI	5	+ 0	−	k.A.	19	
Cannabis-Öl [22] *n* = 30 Endpunkt: CMAI	−2	+	3 Monate	k.A.	15	
Decken [11] *n* =3 Endpunkt: Vokalisationsdauer	−1	+	10 min	k.A.	11	
Audiotapes [24] *n* = 13 Endpunkt: Verhaltensänderung	−1	+	10 Tage	k.A.	13	
Gruppenmusik [25] *n* = 100 Endpunkt: C‑CMAI	2	+	6 Wochen (+1 Monat)	k.A.	14	
Personalisierte Musik [26] *n* = 74 Endpunkt: Verhaltensänderung	−2	+	4 Wochen	k.A.	15	
Therapeutische Berührung [27] *n* = 10 Endpunkt: ABRS	−2	+/0	3 Tage	k.A.	16	
Multisensorische Stimulation [28] *n* = 30 Endpunkt: S‑CMAI	1	+/0	Bis zu 16 Wochen	k.A.	15	
Multisensorische Stimulation [29] *n* = 32 Endpunkt: S‑CMAI	2	+/0	Bis zu 16 Wochen	k.A.	15	
Öle [30] *n* = 38 Endpunkt: PAS, CMAI	5	+/0	4 Wochen	k.A.	17	
Aroma-Handmassage [31] *n* = 72 Endpunkt: CMAI	4	+	4 Wochen	k.A.	14	
Professionelle Schulung [32] *n* = 48 Endpunkt: C‑CMAI	2	+	3 Monate	k.A.	14	
Professionelle Schulung [33] *n* = 306 Endpunkt: CMAI	2	+	20 Wochen (+ 3 Monate)	k.A.	15	
Tiergestützte Therapie [34] *n* =1 Endpunkt: Verhaltensänderung	−2	+	3 Monate	k.A.	–	
Licht [35] *n* = 60 Endpunkt: C‑CMAI	4	+	10 Wochen	Schwindel, Blendung	17	
EKT [8] *n* =1 Endpunkt: Verhaltensänderung	−2	+	4 Wochen	k.A.	–	
EKT [36] *n* =1 Endpunkt: Verhaltensänderung	−2	+	4 Wochen	k.A.	–	
EKT [37] *n* = 16 Endpunkt: PAS	−2	+	–	Verwirrtheit, Gangstörungen	15	

*ABRS* Agitated Behavior Rating Scale, *C‑CMAI* Chinesischer Cohen-Mansfield Agitation Inventory, *CMAI* Cohen-Mansfield Agitation Inventory, *CMAI‑K* Koreanischer Cohen-Mansfield Agitation Inventory, *EKT* Elektrokonvulsionstherapie, *NPI* Neuropsychiatric Inventory, *PAS* Pittsburgh Agitation Scale, *S‑CMAI* Spanischer Cohen-Mansfield Agitation Inventory

## Diskussion

Sowohl medikamentöse als auch nichtmedikamentöse Interventionen können zu einer leichten Verbesserung von repetitiven Vokalisationen bei Demenzerkrankten führen. Pharmakologische Interventionen sind jedoch häufiger mit Nebenwirkungen behaftet. Die erzielten Effekte sind mit Ausnahme der EKT und der Schulungen von Pflegenden jedoch häufig nur vorübergehend und nicht nachhaltig bzw. sie können aufgrund der kurzen Studiendauer nicht längerfristig belegt werden. Dies ist vor dem Hintergrund der Belastungen der Betroffenen und Angehörigen oder des pflegenden Personals unbefriedigend, da der Leidensdruck enorm sein kann [8, 9]. Aufgrund der Tatsache, dass die Prävalenz demenzieller Erkrankungen deutlich zunimmt und damit auch die damit verbundenen Verhaltensstörungen, besteht hier ein erhöhter Handlungsbedarf [3, 5–7]. Vor dem Hintergrund vergleichbarer Effektivität bei geringerem Nebenwirkungsprofil ist die Anwendung nichtmedikamentöser Verfahren den pharmakotherapeutischen Interventionen vorzuziehen. Dies ist jedoch aufgrund von Personalmangel und den meist höheren (Personal)-Kosten nicht immer möglich. Daher wären effektive medikamentöse Therapieoptionen wünschenswert, die Datenlage ist jedoch insgesamt sehr schwach und lässt keine eindeutigen Empfehlungen zu.

Bei den nichtpharmakologischen Interventionen erwiesen sich insbesondere Musiktherapie, Aromatherapie und professionelle Schulungen als wirksam. Schulungen könnten schnell durchgeführt und damit im Vergleich zu anderen Therapien leichter implementiert werden. Sie wirken nachhaltig bis zu 3 Monate, mutmaßlich auch länger. Die EKT zeigte sich ebenso wirksam, ist jedoch aufgrund der meist fehlenden Einwilligungsfähigkeit der Proband:innen sowie des Aufwandes und der Nebenwirkungen nicht einfach zu implementieren und daher nur bei ausgeprägter und therapieresistenter Symptomatik empfehlenswert.

Bei den pharmakologischen Interventionen waren sowohl Antidepressiva, insbesondere SSRIs, aber auch Pregabalin und Risperidon mäßig wirksam. Diese Pharmaka könnten durchaus als individueller Behandlungsversuch initiiert werden, wobei Antidepressiva aufgrund der Nebenwirkungsprofile zu bevorzugen wären. Bei Antipsychotika hingegen ist Vorsicht geboten. So konnte in einer Fall-Kontroll-Studie mit über 46.000 Proband:innen festgestellt werden, dass insbesondere Antipsychotika bei Patient:innen mit Demenz eine erhöhte Mortalitätsrate aufweisen [38]. Es wurde die number needed to harm (NNH) für verschiedene Antipsychotika, die bei Patient:innen mit Demenz eingesetzt wurden, ermittelt [38]. Die höchste Mortalitätsrate zeigte dabei Haloperidol (3,8 %, NNH 26), gefolgt von Risperidon (3,7 %, NNH 27), Olanzapin (2,5 %, NNH 40) und Quetiapin (2,0 %, NNH 50) [38]. Günstiger schnitten Antidepressiva mit einer NNH von 166 ab [38]. In einer weiteren Studie wurde hervorgehoben, dass Antipsychotika der ersten Generation mit einer höheren Mortalität aufgrund von Schlaganfällen (6,7 %), Hüftfrakturen (6,6 %), Myokardinfarkt (3,5 %) und ventrikulärer Arrhythmie (0,9 %) assoziiert waren im Vergleich zu Antipsychotika der zweiten Generation [39]. Vorerkrankungen wie Diabetes, Herzerkrankungen und zerebrovaskuläre Erkrankungen erhöhen unabhängig voneinander das Sterblichkeitsrisiko bei Einnahme von Antipsychotika [39]. Antidepressiva scheinen bei vergleichbarer Wirkung auf Agitation sicherer zu sein [40]. Pregabalin wiederum führt häufig zu Müdigkeit und Schwindel, was wiederum mit einem erhöhten Sturzrisiko einhergehen kann. Die Auswahl einer Medikation sollte daher auch Vorerkrankungen sowie polypharmazeutische Aspekte einbeziehen.

Ein Review aus dem Jahr 2022 über nichtpharmakologische Interventionen bei disruptiven Vokalisationen schlussfolgert, dass Musik, sensorische Stimulation und Badetherapien zu einer Verbesserung dieser führen, Massagen jedoch eine Zunahme bedingen können [15]. Ein anderes Review sieht auch verhaltenstherapeutische Interventionen und musikalische Aktivierung als mögliche Therapiestrategien [41]. Bezüglich pharmakologischer Interventionen schlussfolgerte ein systematisches Review, dass medikamentöse Strategien individuell wirksam und unwirksam sein können [42]. Es bedarf vor allen Dingen mehr placebokontrollierter Studien, welche sich explizit den repetitiven Vokalisationen bei Demenzerkrankten widmen und nicht zusammengefasst verschiedene Verhaltensstörungen untersuchen. Eine abschließende, weitreichende Datensammlung könnte eine große Bereicherung für die Therapie betroffener Patient:innen und involvierter Personen sein.

## Limitationen

Das Review weist einige Limitationen auf. Zum einen wurden die Auswahlkriterien aufgrund der mangelnden Studienlage sehr weit gefasst. Der Begriff der repetitiven Vokalisation ist oft ungenau definiert und wird in vielen Fällen umschrieben, teilweise mit stark abweichender Bedeutung. Des Weiteren kann es zur Verzerrung des Gesamtergebnisses durch die Auswahl von wissenschaftlichen Arbeiten gekommen sein, die explizite Ergebnisse, meistens verbunden mit einer Verhaltensbesserung, zum untersuchten Sachverhalt hervorgebracht haben. Somit ist ein Bias in Richtung positiver Ergebnisse anzunehmen. Weiterhin ist keine Berechnung der Inter-Rater-Reliabilität erfolgt.

Hinsichtlich der Studienqualität, gemessen mittels Jadad-Score, wurde aufgrund des Einschlusses vieler Fallberichte und unverblindeter, nicht kontrollierter Studien ein Großteil dieser als qualitativ schlecht bewertet. Insgesamt erhielten 20 der 25 eingeschlossenen Artikel weniger als drei Punkte und sind somit als qualitativ schlecht zu bewerten. Der Jadad-Score weist jedoch Schwächen bei der Beurteilung von Kohortenstudien, Fall-Kontroll-Studien und Beobachtungsstudien auf. Auch ist die Bewertung mithilfe der STROBE-Checkliste sehr subjektiv, da nicht immer eindeutig entschieden werden kann, ob ein Item der Liste zutrifft. Dabei erfüllten die in diesem Review inkludierten Studien zwischen 11 und 19 von maximal 22 möglichen Items.

Es wurden einige Fallberichte in das Review mit einbezogen, bei denen es sich lediglich um Einzelfallschilderungen handelt, die nicht repräsentativ sind. Aufgrund der Tatsache, dass das Review insgesamt nur wenige placebokontrollierte Studien beinhaltet sowie viele Studien eine geringe Fallzahl aufweisen, muss stets eine Übertragung der Ergebnisse auf ein reales Szenario kritisch hinterfragt werden. Aus diesem Grund und der Heterogenität der eingeschlossenen Studien konnte keine Metaanalyse aus den Daten erstellt werden.

Mit Rücksicht auf die Proband:innen ist zu vermerken, dass alle Subtypen der Demenzerkrankung mit unterschiedlicher Ausprägung einbezogen wurden. Diese wiesen unterschiedliche somatische Vorerkrankungen auf und wurden häufig polypharmazeutisch behandelt. Schließlich ereignet sich die verbale Agitation häufig zufällig und spontan und es kann nicht zwingend eine Kausalität zwischen erfolgter Intervention und einer Verhaltensänderung hergestellt werden.

Nichtsdestotrotz bietet dieses Review einen wissenschaftlichen Überblick über die aktuellen Therapieoptionen eines zunehmend relevanten Themas.

## Conclusio

Sowohl medikamentöse als auch nichtmedikamentöse Interventionen können zu einer leichten Verbesserung von repetitiven Vokalisationen bei Demenzerkrankten führen. Pharmakologische Interventionen sind jedoch häufiger mit Nebenwirkungen behaftet und sollten daher erst nach nichtpharmakologischen Interventionen und individueller Abwägung der Risiken eingesetzt werden. Medikamentöse als auch nichtmedikamentöse Effekte sind mit Ausnahme der EKT sowie der professionellen Schulung von Pflegenden in der Regel nur vorübergehend und nicht nachhaltig bzw. es fehlen Daten zur Langzeitbehandlung. Dies ist vor dem Hintergrund der Belastungen der Betroffenen und Angehörigen oder des pflegenden Personals unbefriedigend, da der Leidensdruck enorm sein kann [8, 9]. Aufgrund der Tatsache, dass diese Umstände für eine große Anzahl an Erkrankten zutreffen, ist hier besonderer Handlungsbedarf indiziert [3, 5–7].

## Supplementary Information

Suchstrategien: Ausführliche Auflistung inkludierter Arbeiten

Ergebnisse der Interventionen
