# Supplementary material for: Therapy strategies for repetitive vocalizations in dementia: A systematic review
Source: Neuropsychiatr. 2024 Oct 15;39(4):174–83. [Article in German] doi: 10.1007/s40211-024-00511-5 (PMC12660410; doi:10.1007/s40211-024-00511-5)
Supplement: Supplementary file 1 — Suchstrategien: Ausführliche Auflistung inkludierter Arbeiten [file 40211_2024_511_MOESM1_ESM.docx]

Suchstrategie Review Repetitive Vokalisationen

Bei der Literaturrecherche wurden die zwei Datenbanken MEDLINE (Pubmed) und Embase gewählt, da es sich hierbei um führende medizinische Datenbanken handelt, welche ein breite Auswahl an medizinischer Literatur beinhalten.

Bei der Suche wurden folgende Wörter recherchiert: „Dementia“, „Vocalisation“, „Repetitive Vocalisation“, „verbal agitation“, „screaming“, „shouting“, „vocally disruptive behaviour“, „noise making“, „verbal agression“, „yelling“, repetitious mannerisms“ und „persistent vocalisation“.

Interventionen (pharmakologisch): antidepressants, antipsychotics, mood stabilizers, benzodiazepines, anxiolytics, stimulants, anti-dementia drugs, acetylcholinesterase inhibitors, donepezil

Interventionen (nicht-pharmakologisch): ect, bathing, massage, aroma therapy, music

| Recherdatum und Uhrzeit | Suchbegriff | Suchtrefferanzahl | Ausgewählte Suchtreffer (Hits) |
| --- | --- | --- | --- |
| Pubmed Suche  27.11.2023 | Repetitive vocalisation AND dementia | 17 | 7 |
|  | Vocalisation AND dementia | 295 | 29 |
|  | Verbal agitation AND dementia | 259 | 43 |
|  | „verbal agitation“ AND dementia | 36 | 9 |
|  | Screaming AND dementia | 72 | 14 |
|  | repetitious mannerisms AND dementia | 8 | 3 |
|  | Shouting AND dementia | 37 | 5 |
|  | Noise making AND dementia | 48 | 3 |
| 28.11.2023 | Vocally disruptive behaviour AND dementia | 24 | 7 |
|  | Verbal aggression AND dementia | 292 | 26 |
|  | Yelling AND Dementia | 24 | 4 |
|  | Persistent vocalisation AND Dementia | 11 | 4 |
| Kombination mit Interventionen | Vocalisations AND dementia AND antidepressants | 7 | 2 |
|  | Vocalisations AND dementia AND antipsychotics | 18 | 5 |
|  | Vocalisations AND dementia AND mood stabilizers | 0 | 0 |
|  | Vocalisations AND dementia AND benzodiazepines | 2 | 0 |
|  | Vocalisations AND dementia AND anxiolytics | 1 | 1 |
|  | Vocalisations AND dementia AND stimulants | 0 | 0 |
|  | Vocalisations AND dementia AND anti-dementia drugs | 0 | 0 |
|  | Vocalisations AND dementia AND ect | 3 | 2 |
|  | Vocalisations AND dementia AND bathing | 2 | 1 |
|  | Vocalisations AND dementia AND massage | 1 | 1 |
|  | Vocalisations AND dementia AND aroma therapy | 2 | 1 |
|  | Vocalisations AND dementia AND music | 19 | 3 |
| 29.11.2023 | Verbal agitation AND dementia AND antidepressants | 16 | 5 |
|  | Verbal agitation AND dementia AND antipsychotics | 35 | 7 |
|  | Verbal agitation AND dementia AND mood stabilizers | 2 | 0 |
|  | Verbal agitation AND dementia AND benzodiazepines | 9 | 2 |
|  | Verbal agitation AND dementia AND anxiolytics | 10 | 4 |
|  | Verbal agitation AND dementia AND stimulants | 0 | 0 |
|  | Verbal agitation AND dementia AND anti-dementia drugs | 0 | 0 |
|  | Verbal agitation AND dementia AND ect | 1 | 1 |
|  | Verbal agitation AND dementia AND bathing | 5 | 2 |
|  | Verbal agitation AND dementia AND massage | 6 | 3 |
|  | Verbal agitation AND dementia AND aroma therapy | 6 | 6 |
|  | Verbal agitation AND dementia AND music | 22 | 9 |
| Embase (Ovid) Suche  29.11.2023 | Repetitive vocalisation AND dementia | 4 | 2 |
|  | Vocalisation AND dementia | 176 | 12 |
|  | Verbal agitation AND dementia | 318 | 21 |
|  | repetitious mannerisms AND dementia | 55 | 5 |
|  | Noise making AND dementia | 37 | 5 |
|  | Vocally disruptive behaviour AND dementia | 37 | 9 |
|  | Persistent vocalisation AND Dementia | 4 | 1 |
| 30.11.2023  Kombination mit Interventionen | Vocalisations AND dementia AND antidepressants | 35 | 5 |

|  | Vocalisations AND dementia AND antipsychotics | 25 | 7 |
| --- | --- | --- | --- |
|  | Vocalisations AND dementia AND mood stabilizers | 2 | 0 |
|  | Vocalisations AND dementia AND benzodiazepines | 21 | 2 |
|  | Vocalisations AND dementia AND anxiolytics | 3 | 0 |
|  | Verbal agitation AND dementia AND stimulants | 6 | 0 |
|  | Verbal agitation AND dementia AND anti-dementia drugs | 0 | 0 |
|  | Vocalisations AND dementia AND ect | 3 | 0 |
|  | Vocalisations AND dementia AND bathing | 7 | 0 |
|  | Vocalisations AND dementia AND massage | 7 | 1 |
|  | Verbal agitation AND dementia AND aroma therapy | 3 | 0 |
|  | Vocalisations AND dementia AND music | 30 | 3 |
|  | Verbal agitation AND dementia AND antidepressants | 56 | 5 |
|  | Verbal agitation AND dementia AND antipsychotics | 64 | 7 |
|  | Verbal agitation AND dementia AND mood stabilizers | 13 | 1 |
|  | Verbal agitation AND dementia AND benzodiazepines | 36 | 3 |
|  | Verbal agitation AND dementia AND anxiolytics | 14 | 2 |
| 2.12.2023 | Verbal agitation AND dementia AND stimulants | 6 | 0 |
|  | Verbal agitation AND dementia AND anti-dementia drugs | 0 | 0 |
|  | Verbal agitation AND dementia AND ect | 14 | 3 |
|  | Verbal agitation AND dementia AND bathing | 51 | 15 |
|  | Verbal agitation AND dementia AND massage | 31 | 15 |
|  | Verbal agitation AND dementia AND aroma therapy | 3 | 1 |
|  | Verbal agitation AND dementia AND music | 81 | 22 |
| 22.04.2024 | Acetylcholinesterase inhibitor AND dementia AND verbal | 99 | 2 |
|  | Acetylcholinesterase inhibitor AND dementia AND CMAI | 9 | 0 |
|  | Acetylcholinesterase inhibitor AND dementia AND shouting | 2 | 0 |
|  | Acetylcholinesterase inhibitor AND dementia AND repitition | 14 | 3 |
|  | Acetylcholinesterase inhibitor AND dementia AND repetitive | 21 | 0 |
|  | Donepezil AND Dementia AND verbal | 42 | 0 |
|  | Donepezil AND Dementia AND vocal | 1 | 0 |
|  | Donepezil AND Dementia AND repetitive | 8 | 0 |
|  | Donepezil AND Dementia AND repetition | 7 | 1 |
| Summe |  | 2635 | 362 |

Vorauswahl der Studien

| Studienname | Vorauswahl weil? (Kriterien) | Einschluss? |
| --- | --- | --- |
| Valproate in the  Treatment of Behavioral  Agitation in Elderly  Patients With Dementia | - “Behavioral agitation” - 1995 - Intervention mit Valproat | - Nein, da Definition nicht genau - Behavioral agitation beinhaltet auch physische Vorgänge wie umherwandern oder versuch, die Einrichtung zu verlassen |
| Environmental "White Noise": An Intervention for  Verbally Agitated Nursing Home Residents | - 1996 - Exakte Definition - Intervention Audiotapes | Ja/nein  Definition:  As used by most researchers,  this term includes an array of vocal behaviors including  screaming, calling out, repetitive statements, and moaning   - Wenn dies zutreffend, dann ja! |
| Behavioural symptoms of dementia in  residential settings: A selective review of non-  pharmacological interventions | - Englische Sprache - 2010 - Definition: aggression, screaming,   restlessness, agitation, wandering, culturally inap-  propriate behaviours, sexual disinhibition, hoarding,  cursing and shadowing’ | Definition exakt genug? |
| Effecs of non-pharmacological interventions on disruptive vocalization in nursing home patients with dementia -a systematic review | - Englische sprache - 2022 - Review - Interventionen zusammengefasst | Ja |
| The Noisy Elderly Patient:  Prevalence, Assessment, and  Response to the  Antidepressant Doxepin | - Englisch - 1991 - Interventionen | Nein, da generell von „noisy“ patients gesprochen wird mit diversen Verhaltensweisen |
| Effects of Individualized Music on  Confused and Agitated Elderly Patients | - Englisch - 1993 - Interventionen bezüglich Agitation | Eventuell ja,   - Je nach Definitionslage allerdings nur für 2 Patienten |
| THE USE OF RISPERIDONE IN SEVERELY  DEMENTED PATIENTS WITH PERSISTENT  VOCALIZATIONS | - Englisch - 1997 - Case reports | Ja,   - Expliziter Bezug zur repetitiven Vokalisation |
| Continuous Screaming Controlled by  Electroconvulsive Therapy: A Case Study | - Englisch - 1994 - Case report | Ein Case Report ausreichend? |
| The use of weighted blankets as a novel approach for treatment of  persistent vocalizations in late stage dementia | - 2021 - Intervention mit Gewichtsdecken | Ja,   - Expliziter auf die repetitive Vokalisation - Exakte Definition - Intervention mit Verhaltensänderung |
| ‘Socialised care futility’ in the care of older people in hospital who  call out repetitively: An ethnographic study | - 2020 - Explizite Definition der repetitiven Voksalisation | Nein,   - Genereller Einschluss von kognitiv beeinträchtigten Patienten oder Diagnose von Delir liegt vor - Demenz kann vorliegen, ist aber nicht das einzige Einschlusskriterium |
| Verbal and physical non-aggressive agitated behaviors in  elderly persons with dementia: robustness of syndromes | - 2004 - Unterteilung in vokale und physische Vokalisation | Nein,   - keine Intervention bezüglich der Vokalisation, nur Zuordnung zu Persönlichkeitscharakteristika wie Alter oder Bildung |
| Music and dementia | - 2015 - In extra Abschnitt auf behavioral agitation eingegangen | Nein,   - Keine eigene Ergebnisdarstellung - Nur Verweis auf andere Studien -> diese dann vielleicht interessant |
| Screaming, shrieking and muttering: the noise-  makers amongst dementia patients | - 2002 - Untersuchung von rep. Vokalisation in Abgrenzung von anderem Verhalten | Nein,   - Untersuchung von anatomischen Korrelaten Bildgebung bei rep. Vokalisation |
| Behavioral and psychological symptoms of dementia in elderly demented subjects, long lasting use of AA | - 2007 - Intervention bezüglich Demenz durchgeführt | Nein,   - Nur Gesamtoutcome - Outcome enthält z.B. auch agressives Verhalten etc. |
| Vocally disruptive behavior (VDB) in the institutionalized elderly: A naturalistic  multiple case report | - 2010 - Intervention medikamentös/nicht-medikamentös bezüglich VDB | Ja/nein   - Fraglich, da 3,5% der Probanden keine Demenz hatten - Nachfragen !!! |
| Essential oils for agitation in dementia [rELOAD]: A pragmatic,  cluster-randomized, placebo-controlled, pilot feasibility trial | - 2021 - Bei allen Patienten liegt Demenzerkrankung vor | Ja/nein   - Verbale subscore - Enthält auch strange noises, comlaining etc. |
| Evaluating the effects of a  physical activity on agitation  and wandering (PAAW)  experienced by individuals  living with a dementia in  care homes | - 2018 - Demenzerkrankung liegt vor | Ja/nein   - Verbale subscore   Enthält auch strange noises, comlaining etc.   - CMAI score |
| Oral thc: cbd cannabis extract in main symptoms of  Alzheimer disease: agitation and weight loss | - 2023 - Demenzerkrankung bei allen - Intervention bezühlich verbal agitation | Ja/nein   - Verbale subscore   Enthält auch strange noises, comlaining etc.  CMAI score |
| Management of the Patient  With Disruptive Vocalization | - 1997 - Demenzerkrankung und disruptive Vokalisation | Nein,   - Nur allgemeine Handlungsempfehlungen, keine Untersuchung der Wirksamkeit der Interventionen |
| Management of Verbally Disruptive Behaviors  in Nursing Home Residents | - 1997 - Demenzerkrankung - Intervention bezüglich VDB | Ja/nein   - 97% der Pat. Haben Demenz |
| Assisting Cognitively Impaired Nursing  Home Residents With Bathing: Effects of  Two Bathing Interventions on Caregiving | - 2006 - Demenzerkrankung liegt - Badeintervention | Nein,   - Vor allen Dingen Bezug auf Agression und Agitation, keine explizite Untersuchung der rep. Vokalisation |
| Nonpharmacological Treatment of Agitation:  A Controlled Trial of Systematic  Individualized Intervention | - 2007 - Demenzerkrankung - Pharmakologische und nicht-pharmakologische Interventionen | Nein,   - Nur generell von Agitation gesprochen, keinen expliziten Bezug auf verbale Aktivität |
| A Risk-Benefit Assessment of  Risperidone for the Treatment of  Behavioural and Psychological  Symptoms in Dementia | - 2000 - Demenzerkrankung - Intervention mit Risperidon | Nein,   - BPSD mit weiteren Verhaltensweisen wie wandering oder biting |
| Behavioural Problems Associated  With Dementia - The Role of Newer Antipsychotics | - 1999 - Demenzerkrankung - Interventionen mit Antipsychotika | Nein,   - Keinen genauen Effekt auf repetitive Vokalisation beschrieben |
| Risperidone  A Review of its Use in the Management of the Behavioural  and Psychological Symptoms of Dementia | - 2000 - Demenzerkrankung - Interventionen mit Antipsychotika | Nein,   - BEHAVE-AD score hat keinen expliziten Bezug zur repetitiven Vokalisation |
| Divalproex Sodium for the Treatment  of Behavioural Problems Associated  With Dementia in the Elderly | - 2006 - Intervention mit Natrium Valporat bei demenz | Nein,   - Verbaler subscore nicht genau aufgegliedert   Enthält auch strange noises, comlaining etc. |
| Calming Music and Hand Massage  With Agitated Elderly | - 2002 - Demenz - Musik, Hand massage | Ja/nein   - Verbale subscore   Enthält auch strange noises, comlaining etc.  CMAI score |
| Pain, agitation, and behavioural problems in people  with dementia admitted to general hospital wards:  a longitudinal cohort study | - 2015 - Demenzerkrankung | Nein,   - Assoziation zwischen schmerz und BPSD untersucht |

| Behavioral and psychological symptoms in Patients with dementia as a target for pharmacotherapy with Risperidone | - 2004 - Demenzerkrankungm mit Risperidon behandelt | Wahrscheinlich ja,   - Explizit Änderung bei repetitive mannerism - Allerdings auch anderes Verhalten bei CMAI Score |
| --- | --- | --- |
| Nonpharmacologic Interventions for  Inappropriate Behaviors in Dementia | - 2001 - Nicht-pharmakologische Interventionen | Wahrscheinlich ja,   - Viele Unterschiedliche Ergebnisse - Allerdings Review – Wie genau diese Ergebnisse einbeziehen? |
| Effect of Light on Agitation in  Institutionalized Patients With  Severe Alzheimer Disease | - 2003 - Demenz - Intervention mit Licht | Ja/nein   - BRS: Agitated Behavior Rating Scale - Auf verbale Verhaltensweisen geachtet, aber noch anderes beinhaltet |
| A Comparison of Two Treatments of  Agitated Behavior in Nursing Home  Residents With Dementia: Simulated Family  Presence and Preferred Music | - 2006 - Demenz - Interventionen wie simulated family presence oder music | Ja/nein   - CMAI   Auf verbale Verhaltensweisen geachtet, aber noch anderes beinhaltet |
| Safety and Efficacy of Electroconvulsive  Therapy for the Treatment of Agitation  and Aggression in Patients With Dementia | - 2012 - Demenzerkrankung - Ect intervention | Eher Ja   - Aberrant Vocalization   (repetitive requests or complaints, nonverbal  vocalizations, e.g., moaning, screaming) nach PAS-Score   - Auch nochmal separat aufgeführt |
| Intonational Patterns of Nonverbal  Vocalizations in People With Dementia | - 2011 - Demenzerkrankung - Vokalisation | Nein,   - Vokalisation an sich untersucht - Jedoch keine Intervention mit Änderung |
| Effects of Animal-Assisted Therapy on  Behavioral and/or Psychological Symptoms  in Dementia: A Case Report | - 2012 - Demenzerkrankung - Interventionen mit Tier | Ja/nein   - CMAI   Auf verbale Verhaltensweisen geachtet, aber noch anderes beinhaltet |
| Managing Agitation Using  Nonpharmacological Interventions  for Seniors With Dementia | - 2013 - Demenz - Nicht-pharmkologische Interventionen | - Kein expliziter Bezug auf repetitive Vokalisation |
| Effects of Multisensory Stimulation  on a Sample of Institutionalized Elderly  People With Dementia Diagnosis:  A Controlled Longitudinal Trial | - 2014 - Demenz - Interventionen | Ja/nein   - Verbally non-aggressive behaviour (com-   plaining, constant requests for attention, negativism, repetitious  sentences or questions, and screaming). |
| Multisensory Stimulation as an  Intervention Strategy for Elderly  Patients With Severe Dementia:  A Pilot Randomized Controlled Trial | - 2015 - Demenz - Interventionen | Ja/nein   - Verbally non-aggressive behaviour (com-   plaining, constant requests for attention, negativism, repetitious  sentences or questions, and screaming). |
| Vocalization in Dementia: A Case  Report and Review of the Literature | - 2014 - Demenz - Case Report mit Verhaltensintervention | Ja,   - Intervention mit Verhaltensänderung bezüglich Vokalisation |
| Efficacy of Blue LED Phototherapy on Sleep  Quality and Behavioral and Psychological  Symptoms of Dementia: A Double-Blind  Randomized Controlled Trial | - 2023 - Demenz - Intervention mit Licht | Ja/nein (CMAI)   - Verbally non-aggressive behaviour (com-   plaining, constant requests for attention, negativism, repetitious  sentences or questions, and screaming). |
| Antipsychotics in dementia: use  only if the risks are justified | - 2009 - Demenzerkrankung - Antipsychotikum | Nein,   - Keine Intervention nur Warnhinweis |
| Simulated presence therapy for dementia (Review) | - 2017 - Demenz - Diverse Intervention | Ja,   - Veränderung bezüglich VDB und rep. Vokalisation |
| Simulated presence therapy for dementia (Review) | - 2020 - Demenz - Diverse Intervention | Ja,   - Veränderung bezüglich VDB und rep. Vokalisation |
| Continuum of care: Stabilizing the acutely  agitated patient | - 2002 - Medikamentöse Intervention bei Agitation | Nein,   - Agitation nicht genauer beschrieben und auch auf Schizophrenie bezogen |
| Repetitive and Stereotypic  Vocalization in Dementia after Using  Antipsychotics | - 2021 - Case Report Rep. Vokalisation - Behandlung mit Antipsychotikum | Nein,   - Rep. Vokalisation tritt medikamenteninduziert auf |
| Clinical pathways for the evidence-based management of  behavioural and psychological symptoms of dementia in a  residential aged care facility: A rapid review | - 2021 - Untersuchung bezüglich BPSD | Nein,   - BPSD enthält mehr als rep. Vokalisation |
| ECT Treatment for Two  Cases of Dementia-Related  Pathological Yelling | - 2008 - Case Report bezogen auf Demenz - Besserung im Verhalten | Ja/nein   - Verhaltensänderung im Bezug auf vokalisation - Allerdings nur Case beschreibung |
| Behavioral interventions for agitation  in older adults with dementia:  an evaluative review | - 2006 - Review im Bezug auf unterschiedliches Verhalten bei Demenz | Ja/nein   - Disruptive vocalization (DV) includes vocal behaviors that are repetitive,   disruptive, or otherwise inappropriate (Cohen-Mansfield and Werner, 1994). |
| Behavioural symptoms of dementia in  residential settings: A selective review of non-  pharmacological interventions | - 2010 - Review bei Demenz in Bezug auf BPSD | Nein,   - BPSD und Agitation enthalten noch weiteres Verhalten |
| Managing dementia agitation  in residential aged care | - 2010 - Interventionen in Bezug auf Agitation bei Demenz | Nein,   - Agitation beinhaltet weitere Verhaltensweise - Wird auch im Artikel kritisiert, dass z.B. nicht explizit auf rep. Vokalisation eingegangen wird |
| Positive Interactive  Engagement (PIE): A pilot  qualitative case study  evaluation of a person-centred  dementia care  programme based on  Montessori principles | - 2020 - Interventionen in Bezug auf Agitation bei Demenz | Nein,   - Agitation beinhaltet weitere Verhaltensweise |
| Screaming in elderly persons  with dementia | - 2008 - Screaming bei Demenz behandelt | Ja,   - Laut genannter Definition müsste es rep. Vokalisation entsprechen |
| Massage and touch for dementia (Review) | - 2006 - Intervention bei Demenz | Nein,   - Kein Bezug zur rep. Vokalisation |
| Optimal nonpharmacological management of  agitation in Alzheimer’s disease: challenges and  solutions | - 2016 - Interventionen bei Agitation bei Demenz | Ja/nein   - CMAI |
| Antipsychotics for the Treatment of Behavioral and Psychological  Symptoms of Dementia (BPSD) | - 2018 - Interventionen bei BPSD bei Demenz | Nein,   - BPSD nicht genauer klassifiziert |
| Vocally disruptive behaviour in nursing home residents in  Ireland: a descriptive study | - 2020 - Interventionen bei VDB | Nein,   - Demenz nicht notwendiges Einschlusskriterium |
| Effectiveness of Montessori-based activities on  agitation among Asian patients with dementia  A systematic review and meta-analysis | - 2022 - Interventionen bei Agitation bei Demenz | Nur Ja,  wenn verbal non-agressive behaviour nach CMAI erlaubt ist |
| Ethical dilemmas: should antipsychotics ever  be prescribed for people with dementia? | - 2010 - Interventionen mit Antipsychotika bei Demenz | Nein,   - Nur allgemeine Handlungsempfehlungen im Umgang mit Antipsychotika bei Demenz |
| Aromatherapy for dementia (Review) | - 2014 - Intervention bei Demenz bezüglich Verhalten | Nur Ja,  wenn verbal non-agressive behaviour nach CMAI erlaubt ist |
| Effects of nonpharmacological interventions on disruptive vocalization in nursing home patients with dementia – a systematic review | - 2022 - Nicht-pharmakologische interventionen bei disruptive behaviour bei demenz | Ja,   - Explizit mit Wirksamkeit aufgelistet |
| The Noisy Elderly Patient:  Prevalence, Assessment, and  Response to the  Antidepressant Doxepin | - 1992 - Pharmakologische Intervention bei Demenz mit Verhaltensänderung | Eher ja,   - Wenn screaming etc. ingesamt zur rep. Vokalisation zählt |
| Factors Associated with Problematic  Vocalizations in Nursing Home Residents  With Dementia | - 2021 - Vokalisationen bei Demenz | Nein,   - Nur assoziierte Faktoren bei diesem Verhalten untersucht |
| Influence of aromatherapy on medication  administration to residential-care residents  with dementia and behavioral challenges | - 2002 - Aromatherapie bei behavioural challenges bei Demenz | Nein,   - Nur von resistive Behaviours gesprochen, nicht weiter erläutert |
| Therapeutic Touch and  Agitation in Individuals  With Alzheimer’s Disease | - 2008 - Therpeutic Touch bei Demenz | Nur Ja,  wenn verbal non-agressive behaviour nach CMAI erlaubt ist |
| Effects of Music in Reducing Disruptive  Behavior in a General Hospital | - 2006 - Interventionen mit Musik in Bezug auf Disruptive Behaviour | Nein,   - Nicht zwingend Demenzerkrankung notwenig - Keinen explziten Bezug zur rep. Vokalisation |
| Favorite music and hand  massage  Two interventions to decrease agitation in residents  with dementia | - 2008 - Interventionen bezüglich Agitation bei Demenz | Nur Ja,   - wenn verbal non-agressive behaviour nach CMAI erlaubt |
| Aroma therapy for dementia (Review) | - 2010 - Interventionen bei Demenz bezüglich Verhalten | Nur Ja,   - wenn verbal non-agressive behaviour nach CMAI erlaubt |
| THE EFFICACY AND TOLERABILITY OF  DIVALPROEX SODIUM IN ELDERLY DEMENTED  PATIENTS WITH BEHAVIORAL DISTURBANCES | - 1998 - Interventionen bei Demenz bezüglich behavioral disturbances | Nur Ja,   - wenn verbal non-agressive behaviour nach CMAI erlaubt |
| A pilot study on a home-based caregiver training program for  improving caregiver self-efficacy and decreasing the  behavioral problems of elders with dementia in Taiwan | - 2003 - Intervention bei Demenz bezüglich behavioral problems | Nur Ja,   - wenn verbal non-agressive behaviour nach CMAI erlaubt |
| Opioid treatment for agitation in patients with advanced  Dementia | - 2003 - Interventionen bezüglich Agitation bei Demenz | Nein,   - CMAI nur allgemein und nicht genauer unterteilt |
| The management of inappropriate vocalisation  in dementia: a hierarchical approach | - 2005 - Interventionen bezüglich Vokalisation bei Demenz | Eher nein,   - Nur Studiennennung aus anderen Kontexten - Eventuell, wenn Sekundärliteratur zitiert werden kann |
| Comparative efficacy of risperidone versus haloperidol on  behavioural and psychological symptoms of dementia | - 2006 - Interventionen bei Demenz bezüglich psychologischen Symptomen | Ja,   - Expliziter Bezug zur rep. Vokalisation |
| Expert opinion on the management of behavioural and  psychological symptoms of dementia (BPSD) and  investigation into prescribing practices in the UK | - 2009 - Management von psychologischen Symptomen von Demenz | Nein,   - Meinungsbild nach Expertenumfragen - Keine direkten Interventionen in Bezug auf rep. Vokalisation |
| Non-pharmacological management of behavioural  symptoms in nursing homes | - 2009 - Management von Verhaltenssymptomen bei Demenz | Nur Ja,   - wenn verbal non-agressive behaviour nach CMAI erlaubt |
| Effectiveness of group music intervention against agitated  behavior in elderly persons with dementia | - 2010 - Demenzerkrankung und Musikinterventionen mit Verhaltensänderung | Nur Ja,   - wenn verbal non-agressive behaviour nach CMAI erlaubt |
| The effect of music therapy compared with general  recreational activities in reducing agitation in people with  dementia: a randomised controlled trial | - 2012 - Demenzerkrankung und Musikinterventionen mit Verhaltensänderung | Nur Ja,   - wenn verbal non-agressive behaviour nach CMAI erlaubt |
| Comparison of the efficacy of gesture‐verbal treatment and  doll therapy for managing neuropsychiatric symptoms in older  patients with dementia | - 2018 - Management von neuropsychiatrischen Symptomen bei Demenz | Nein,   - Bezug auf NPI-Score, der nicht explizit rep. Vokalisation beinhaltet |
| Phenomenological contribution to understanding of vocally  disruptive behaviour: A clinical case study in a patient with  dementia | - 2018 - Behandlung von VDB | Nein,   - VDB beinhaltet auch aggressives Verhalten und keine spezifische Intervention mit Verhaltensänderung |
| Why deprescribing antipsychotics in older people with  dementia in long‐term care is not always successful: Insights  from the HALT study | - 2019 - BPSD Behandlung bei Demenz | Nein,   - Bezug auf NPI-Score, der nicht explizit rep. Vokalisation beinhaltet - Demenzdiagnose nicht explizit notwendig |
| Prevalence and correlates of psychotropic drug use in Dutch  nursing home patients with young‐onset dementia | - 2019 - Psychotrope Medikation bei Demenz | Nein,   - nur Assoziation von psychoaktiven Substanzen zum verbal agitiertem Verhalten |
| Continuous Screaming Controlled by  Electroconvulsive Therapy: A Case Study | - 1994 - EKT bei dauerhaftem Schreien bei Demenz | Ja,   - Explizite Intervention mit Veränderung von rep. Schreien |
| Individualized music played for agitated patients  with dementia: Analysis of video-recorded sessions | - 2001 - Musikintervention bei Agitation bei Demenz | Nein,   - Generell von Agitation gesprochen ohne genauere Bezeichnung |
| Tactile stimulation associated with nursing care to individuals with  dementia showing aggressive or restless tendencies: an intervention  study in dementia care | - 2006 - Taktile Stimulation bezüglich Verhalten bei Demenz | Nein,   - Hauptsächlich Bezug auf BPSD und Aggressionen |
| What are the relative merits of interventions used to reduce the  occurrences of disruptive vocalisation in persons with dementia? – a  systematic review | - 2015 - Interventionen, um disruptive Vokalisationen bei Demenz zu reduzieren | Nur Ja,   - wenn verbal non-agressive behaviour nach CMAI erlaubt |
| Nursing home residents with advanced dementia and  persistent vocalisations: Observations of surrounding context | - 2020 - Interventionen bei rep. Vokalisation bei Demenz | Ja,   - Expliziter Bezug zur rep. Vokalisation |
| Integrative review: Persistent vocalizations among nursing  home residents with dementia | - 2019 - Artikel über persistierende Vokalisationen bei Demenz | Nein,   - Keine konkreten Daten zur Veränderung von rep. Vokalisation nach Intervention |
| Withdrawal of Haloperidol, Thioridazine,  and Lorazepam in the Nursing Home | - 1999 - Medikamentöse Intervention bezüglich verbaler Agitation | Nein,   - Demenz kein notwendiges Einschlusskriterium |
| Effectiveness of Nonpharmacological Interventions  for the Management of Neuropsychiatric Symptoms  in Patients With Dementia | - 2006 - Nichtpharmakologische Intervention bei Demenz bezüglich neuropsychiatrischer Symptome | Nein,   - Verble Agitation nicht genauer erläutert |
| Assessment and Treatment of Nursing Home Residents with  Depression or Behavioral Symptoms Associated with Dementia:  A Review of the Literature | - 2003 - Behandlung von Verhaltenssymptomatik von Patienten mit Demenz | Eventuell ja,   - Muss genau geschaut werden, was unter verbaler Agitation verstanden werden kann |
| Effect of the Bathing Without a Battle Training Intervention on  Bathing-Associated Physical and Verbal Outcomes in Nursing  Home Residents with Dementia: A Randomized Crossover  Diffusion Study | - 2014 - Baden als Intervention bei verbaler Agitation bei Demenz | Nein,   - Verbesserung des verbalen Verhaltens nicht genau beschrieben (ob z.B. mit rep. Vokalisation übereinstimmt) |
| Severe Disruptive Vocalizers | - 1999 - Interventionen und Behandlungsvorschläge bei disruptiver Vokalisation | Nein,   - 18% der Probanden und Probandinnen hatten keine Demenz-Diagnose |
| FUNCTIONAL ASSESSMENT AND NONCONTINGENT  REINFORCEMENT IN THE TREATMENT OF  DISRUPTIVE VOCALIZATION IN ELDERLY DEMENTIA PATIENTS | - 2002 - Interventionen bei disruptiver Vokalisation bei Demenz | Ja,   - Verhaltensänderung explizit bei disruptiver Vokalisation |
| The effect of massage on agitated behaviours in older people with  dementia: a literature review | - 2012 - Interventionen bei Agitation bei Demenz mit Verhaltensänderung | Nein,   - Ergebnisse nicht genau untergliedert bezüglich verbalen (nicht-aggressiven Verhaltens) |
| Sensory stimulation for persons with dementia: a review of the  Literature | - 2016 - Sensorische Stimulation bei Patienten mit Demenz | Nein,   - Keine explizite Aufgliederung der Agitation oder BPSD nach verbalem Verhalten |
| Citalopram for Verbal Agitation  in Patients with Dementia | - 2000 - Medikamentöse Intervention Gegen verbale agitation bei Demenz | Ja,   - Explizite Definition von verbalem-nicht aggressivem Verhalten mit Verhaltensänderung |
| Verbal Agitation in Dementia: The Role of  Discomfort | - 2010 - Bezug auf verbale Agitation bei Demenz | Nein,   - Ergebnisse für verbale Agitation nicht genau aufgegliedert und eher im Bezug zum Unbehagen der Patienten und Patientinnen |
| Alzheimer disease: Non-  pharmacological and  pharmacological management  of cognition and neuropsychiatric  symptoms | - 2018 - Nicht-medikamentöse und medikamentöse Behandlung neuropsychiatrischer Symptome bei Alzheimer Demenz | Nein,   - Generell von BPSD mit Halluzinationen etc. gesprochen oder verbal-aggressivem Verhalten |
| Haloperidol for agitation in dementia (Review) | - 2012 - Haloperidol für Behandlung von Agitation in Demenz | Ja,   - Aber darauf achten, dass wirklich nur auf verbale Vokalisation bezogen wird |
| Gabapentin for Treatment of Behavioral and  Psychological Symptoms of Dementia | - 2001 - Behandlung psychischer Symptome und von Verhaltenssymptomatik bei Demenz | Nein,   - Insgesamt auf BPSD mit Aggressionen etc. bezogen |
| Music in the nursing home: hitting the right note! The  provision of music to dementia patients with verbal and vocal  agitation in Dutch nursing homes | - 2008 - Musikinterventionen bei verbaler und vokaler Agitation bei demenzerkrankten Personen | Nein,   - Interviews von Ärzten und Ärztinnen und Pflegepersonal über Musikangebot, jedoch keine genauen Untersuchungen bezüglich der Verhaltensänderung |
| Behavior management approach for agitated  behavior in Japanese patients with dementia:  a pilot study | - 2013 - Interventionen bei agitiertem Verhalten bei Demenz | Nein,   - CMAI nicht genau aufgegliedert, nur allgemein (enthält auch aggressive Handlungen etc.) |
| Nonpharmacological and pharmacological  interventions for symptoms in Alzheimer’s disease | - 2014 - Pharmakologische und nicht-pharmakologische Behandlung von Alzheimersymptomen | Nein,   - Keinen expliziten Bezug zur rep. Vokalisation - Nur von BPSD und Agitation im Allgemeinen gesprochen |
| Agitated Behavior in Elderly Nursing Home Residents  With Dementia in Japan | - 2002 - Expliziter Bezug auf Vokalisationen etc. bei Demenz | Nein,   - Nur Auflistung von Häufigkeiten und anderen statistischen Merkmalen, jedoch keine Interventionen mit Verhaltensänderungen |
| A Review of Anticonvulsants in Treating Agitated  Demented Elderly Patients | - 1998 - Antikonvulsiva zur Behandlung von Agitation bei Demenz | Nein,   - Nur von Agitation im allgemeinen gesprochen, auch mit aggressivem Verhalten etc. |
| Pharmacological management of behavioural and psychological  symptoms of dementia | - 2016 - Pharmakologische Interventionen bei psychischer Symptomatik bei Demenz | Nein,   - Nur BPSD allgemein mit z.B. psychotischen Zuständen oder Halluzinationen etc. |
| Managing challenging behaviour in  older adults with dementia | - Jahr? - Management von schwierigem Verhalten bei an Demenz erkrankten Patienten und Patientinnen | Nein,   - Generell nur Behandlungsoptionen aufgeführt ohne |
| Screaming behaviour in response to electroconvulsive  treatment: psychogeriatric note on a patient with Alzheimer’s  disease | - 2019 - Case Report über EKT bei Demenz in Kombination mit rep. Vokalisation | Ja,   - VDB explizit mit repetitiven Vokalisationen definiert |
| Treatment of Verbal Agitation with a  Selective Serotonin Reuptake Inhibitor | - 2000 - Behandlung von verbaler Agitation bei Demenz | Eher ja,   - Expliziter Bezug zu Item 23 CMAI (rep. Sätze) - Allerdings nichmal prüfen, ob wirklich Ergebnisse vorliegen |
| Reducing verbal agitation in people with  dementia: Evaluation of an intervention based on  the satisfaction of basic needs | - 2011 - Reduktion von verbaler Agitation bei Demenz durch Interventionen | Ja,   - Expliziter Bezug zu repetitivem vokalem Verhalten mit Verhaltensänderung nach Intervention |
| Effects of Relaxing Music on Agitation  DuringMeals Among Nursing Home  Residents With Severe  CognitiveImpairmen | - 1994 - Effekten von Entspannungsmusik auf Agitation | Nein,   - Demenz keine erforderliche Einschlussdiagnose, nur generell kognitive Beeinträchtigung |
| “Music First”  An Alternative or Adjunct to Psychotropic Medications for  the Behavioral and Psychological Symptoms of Dementia | - 2018 - Musikinterventionen bei Agitation bei Demenz | Nein,   - CMAI nicht weiter untergliedert |
| Risperidone, Haloperidol, and Olanzapine for the  Treatment of Behavioral Disturbances  in Nursing Home Patients:  A Retrospective Analysis | - 2000 - Behandlung von störendem Verhalten bei Demenz mit medikamentöser Intervention | Nein,   - Allgemein nur auf Verhalten bezogen (hitting, screaming, pacing) |
| Elektrokonvulsionstherapie zur  Behandlung therapieresistenter  Vokalisationen bei Demenz | - 2018 - Behandlung von Vokalisation bei Demenz durch EKT | Ja,   - Expliziter Bezug zur (repetitiven) Vokalisation mit Verhaltensänderung |
| Communication improvement reduces BPSD: a music therapy study  based on artificial neural networks | - 2021 - Musiktherapie bei BPSD bei Demenz | Nein,   - Nur allgemein von BPSD und Agitation gesprochen, nicht genauer auf verbalen Aspekt eingegangen |
| Nonpharmacologic Treatment  of Behavioral Disorders  in Dementia | - 2013 - Nicht-pharmakologische Behandlung von Verhaltensstörungen bei Demenz | Eher nein,   - nur allgemeine Behandlungsergebnisse durch Erkenntnisse aus Reviews etc. z.B. ohne konkreten Verweis auf Daten |
| The outcomes of a person-centered, non-  pharmacological intervention in reducing  agitation in residents with dementia in  Australian rural nursing homes | - 2021 - Nicht-pharmakologische Behandlung zur Reduktion von Agitation bei Demenz | Ja,   - explizite Zahlen zur rep. Vokalisation |
| Benefits of music therapy on behaviour disorders in  subjects diagnosed with dementia: A systematic  review | - 2014 - Musiktherapie bei Verhaltensstörungen bei Demenz | Ja,   - Aber nur ein Unterergebnis CMAI |
| Agitation in dementia and the role of spatial and sensory  interventions: experiences of professional and family  caregivers | - 2015 - Behandlung von Agitation bei Demenz durch räumliche und sensorische Intervention | Nein,   - Nicht genau auf verbalen Aspekt eingegangen und Zusammenfassung von Erfahrungen |
| Sleep and agitation in nursing home residents with and  without dementia | - 2015 - Untersuchung von Agitation bei Demenzpatienten und -patientinnen | Nein,   - Zusammenhang Schlaf und Agitation, aber keine Intervention |
| The place for electroconvulsive therapy in  the management of behavioral and  psychological symptoms of dementia | - 2019 - EKT zur Behandlung von psychologischen Symptomen bei Demenz | Ja,   - Wenn Unterergebnisse verwendet werden, aber nur zur disruptive vocalisation -> Frage der Definition |
| Efficacy and Tolerability of Carbamazepine  for Agitation and Aggression in Dementia | - 1998 - Pharmakologische Intervention Gegen Agitation bei Demenz | Nein,   - BPRS und CGI Score zu weit gefasst, kein expliziten Bezug zur rep. Vokalisation |
| The efficacy of psychosocial approaches to  behaviour disorders in dementia: a systematic  literature review | - 2010 - Psychosoziale Interventionen bei Verhatensstörungen bei Demenz | Eher ja,   - Muss aber geprüft werden, ob Unterpunkt so aus Literaturreview übernommen werden kann |
| Very frequent physical aggression and  vocalizations in nursing home residents with  dementia | - 2020 - Vokalisationen von Heimbewohnern mit Demenz | Nein,   - Zuordnung zu demographischen Zusammenhängen, keine Interventionen |
| Vocally disruptive behaviour in dementia: Development  of an evidence based practice guideline | - 2005 - Behandlungsguide für disruptive Vokalisation bei Demenz | Ja,   - Wenn aus Sekundärliteratur zitiert werden darf |
| Vocally disruptive behavior: A case report and  literature review | - 2022 - Case Report zur Behandlung von repetitiver Vokalisation | Ja,   - Case Report zur expliziten Behandlung von repetitiven Vokalisation |
| Vocally disruptive behavior in the elderly:  a systematic review | - 2008 - Interventionen zur Behandlung von VDB bei Demenz | Eher ja,   - Aber nochmal prüfen, ob wirklich Ergebnis auf Demenz bezogen werden können |
| The Effect of Multisensory Stimulation  on Persons Residing in an Extended  Care Facility | - 2009 - Multisensorische Stimulation bei dementen Patienten | Nein,   - Keinen expliziten Bezug zur rep. Vokalisation |
| Comparative Efficacy of Interventions for Aggressive and Agitated  Behaviors in Dementia | - 2019 - Interventionen bei agressivem und agitiertem Verhalten bei Demenz | Nein,   - Abgrenzung von verbaler Aggression oft nicht möglich bzw. verbale Agitation nicht genug erläutert |
| The Effect of Therapeutic Touch on  Agitated Behavior and Cortisol in  Persons with Alzheimer’s Disease | - 2002 - Therapeutische Berührungen bei Agitation bei Demenz | Ja,   - Aber nur vokalen Aspekt extrahieren |
| The Effect of Aroma Hand Massage Therapy  for People with Dementia | - 2015 - Massage für Menschen mit Demenz | Ja,   - CMAI explizit für repetitive mannersims aufgegliedert |
| Low investment non-pharmacological  approaches implemented for older people  experiencing responsive behaviours of  dementia | - 2020 - Nicht-pharmakologische Interventionen bei responsivem Verhalten bei Demenz | Nein,   - Keine explizite Intervention bezüglich Vokalisation |
