# Supplementary material for: Therapy strategies for repetitive vocalizations in dementia: A systematic review
Source: Neuropsychiatr. 2024 Oct 15;39(4):174–83. [Article in German] doi: 10.1007/s40211-024-00511-5 (PMC12660410; doi:10.1007/s40211-024-00511-5)
Supplement: Supplementary file 2 — Ergebnisse der Interventionen [file 40211_2024_511_MOESM2_ESM.docx]

**Ergebnisse**

**Pharmakologisch**

**Antidepressiva**

| Studie, Erst-autor, Ver-öffent-lichungs-jahr | Anzahl Studien-teilnehmer  (Geschlechts-verteilung ♂♀ und Durchschnitts-alter Ø) | Studien-design | Messinstrument | Intervention | Ergebnis  (Reduktion ↓) | Limitationen und mögliche Neben-wirkungen |
| --- | --- | --- | --- | --- | --- | --- |
| Citalo-pram  Kim et al, 2000  [16] | 2 (♂:2; Ø 60.5) | Fallserie | Verhaltens-änderung | A:  20 mg Citalopram/Tag, Nach Verbesserung: 40mg/Tag  B:  20 mg Citalopram/Tag,  1 Woche später 20mg/2xTag | A: ↓ verbale Agitation nach 10 Tagen  B:  Verhaltensbesserung innerhalb 10 Tage  verbale Agitation ↓80% | A: 5 Tage nach Therapie-abbruch erneute verbale Agitation (Applikations-problem); Agitation kann spontan und zufällig sein ohne identifizierbare Gründe |
| Par-oxetin  Ramadan et al, 2000  [17] | 15 (12 ♀, 3 ♂; Ø79)  Pflegeheimgruppe/  ambulante Gruppe | Offene Fallserie | CMAI  (repetitive Fragen und Sätze Item 23, ständige ungerechtfertigte Bitte um Aufmerksamkeit Item 29) | Paroxetin ein Monat nach Observierung begonnen, 10 mg oral/Tag, in 10 mg - Schritten im 2 Wochen Intervall erhöht, wenn Punktzahl Item 23 oder Item 29 ≥4, maximale Dosis 40 mg pro Tag/zwei geteilte Dosen  Studienende: zwei Proband:innen 40 mg/Tag, 13 Proband:innen 10-30 mg/Tag | Ende 1. Monat: Pflegeheimgruppe:  Punktescore ↓43-57%,  ambulante Gruppe: ↓50-67%  Ende 3. Monat: ↓ 67-71% bei 5 Proband:innen (Baseline)  CMAI ≤ 3 bei 13 Proban:innen (3. Monat) = 87% | Verschlechterung des Parkinson-tremors und Diarrhö, Gewichts-zunahme; Kleine, uneinheitliche Proband:innengruppe ohne Kontrollgruppe; Bewertung unverblindet und Bewertung nur durch Pflegekräfte |

**Antipsychotika**

| Risperidon  Kopala & Honer, 1997  [12] | 2 (2 ♀; Ø 85) | Fallserie | Frequenz der Vokalisationen | A:  Start Risperidon 0.5 mg/2x Tag,  nächtlich 1 mg,  danach 1.5 mg/ Tag für 3 Wochen  B:  0.5 mg Risperidon/ 2x Tag  nächtlich 1 mg | A:  ↓ Woche 1, Vokalisation ↓‹10% reduziert (Baseline) nach 3 Wochen  B:  Nach einer Woche: Frequenz ↓ 50% (Baseline)  Nach 8 Wochen: ↓‹20% | A: innerhalb 1 Woche nach Therapieunter-brechung erneut Vokalisationen; eine Probandin Verbesserung bukkolinguale Dyskinesie |
| --- | --- | --- | --- | --- | --- | --- |
| Risperidon und Haloperidol  Guk-Hee Suh et al, 2006  [18] | 120 (114 bis Versuchsende; 96 ♀, 24 ♂; Ø 80.9) | Post-hoc-Analyse | Koreanischer CMAI  (CMAI-K) | Risperidon/ Haloperidol bei 0.5 mg/Tag begonnen, mögliche Erhöhung alle 4 Tage oder mehr um 0.5 mg-Schritte bis Maximum von 1.5 mg/Tag, Datenerhebung alle 2 Wochen bis 8 Wochen | CMAI-K  Risperidon signifikant effektiver in der Behandlung von repetitiven Sätzen oder Fragen (p=0.0025), Haloperidol bezüglich keines Verhaltens signifikanter Risperidon überlegen | Proband:innengröße eventuell zu klein für Effekte bei individuellen Verhaltensweisen; Haloperidol-Dosis möglicherweise zu gering; Patient:innen-bedingungen möglicherweise nicht auf Baseline-Bedingungen zurückgegangen; negative Effekte auf tägliche Rhythmusstörungen, Schlaflosigkeit und Somnolenz häufiger beim Haloperidol; Haloperidol assoziiert mit Zunahme extrapyramidaler Symptomatik |
| Thioridazin, Haloperidol und Perphenazin  Friedman et al, 1992  [19] | 11 (7 ♀, 4 ♂, Ø 87) | Fallserie | Observierung | Thioridazin, Haloperidol und Perphenazin | 3x Verbesserung, 8x kein Ansprechen, 3x Verschlechterung | Kleine Proband:innen-gruppe; Einzelfall-schilderungen; unverblindet; retrospektiv; keine Dosierungsangaben |

**Antiepileptika/Antikonvulsiva**

| Gabapentin und Pregabalin  McCarthy et al, 2022  [5] | 1 (♀, Ø 88) | Fallbericht | Observierung | 100 mg Gabapentin; (abgesetzt da Nebenwirkungen); danach 50 mg Pregabalin | Gabapentin: ↓Frequenz/Stärke vokal-disruptives Verhalten  Pregabalin: ↓Frequenz/Stärke von Agitation & Schimpfen innerhalb von 2 Tagen; nach 2 Wochen Behandlung ↓Schreien; Verbesserung über 9 Monate aufrecht erhalten | Myoklonisches Zucken nach Gabapentin; nur eine Probandin |
| --- | --- | --- | --- | --- | --- | --- |

**Acetylcholinesteraseinhibitoren/Memantin**

| Donepezil  Asp et al., 2006  [20] | 46  (28 Nicht- Anprechende, Ø74.1 Jahre, ♀ 64.3 % ; 18 Ansprechende, Ø77.7, 72.2 %♀) | Sekundäranalyse der offenen „Atlantic Canada Alzheimer’s Disease Investigation of Expectations“ Studie  52 Wochen Donepezil bei mild-moderater AD | GAS (Goal Attainment Scaling) |  | 18 Ansprechende Probanden nach 9 Monaten, 12 davon nach 6 (67%) und 15 nach 3 Monaten (83%) angesprochen auf Intervention; 15 auch nach 12 Monaten angesprochen (83%) | Sekundäranalyse |
| --- | --- | --- | --- | --- | --- | --- |
| Donepezil vs. Memantin  Lockhart et al, 2011 (Review)  [21] |  | 4 RCT Donepezil  2 RCT Memantin | NPI |  | Statistisch signifikante Ergebnisse Donepezil Monotherapie (milde bis moderate Alzheimerdemenz) im Vergleich zum Placebo (WMD –3.51, 95% CI –5.75, –1.27)  Keine statistisch signifikanten Ergebnisse zwischen Memantin Monotherapie und Placebo (WMD –1.65, 95% CI –4.78, 1.49)  Donepezil gegenüber Memantin leicht begünstigt, Unterschied nicht statistisch signifikant (p= 0.34) |  |

**Cannabinoide/orales THC**

| Orales THC  Palmieri & Vadalà, 2023  [22] | 30 (21 ♀, 9 ♂; Ø 69.36) | retrospektiv, analytisch, beobachtender, spontaner Versuch | CMAI | Mit Öl verdünnter Cannabis- extrakt, Bedrocan® (22% THC,  0.5% CBD, Olivenöl 50 ml), sublingual  2x/ Tag für 12 Wochen; von 15 Tropfen bis maximal 30 Tropfen/ Tag (1ml); Reduktion auf 0.5 ml/Tag im 3. Monat | Einige Verhaltensweisen wie Fragenwiederholung häufiger beobachtet; verbal nicht-aggressives Verhalten Ø 4↓ 2 ; Schreien am Behandlungsende ↓ | Kleine Proband:innengruppe; fehlende Kontrollgruppe; selbstausgefüllte Fragebögen, die nicht objektiv sind |
| --- | --- | --- | --- | --- | --- | --- |

**Nicht pharmakologisch**

**Decken**

| Gewichtsdecken  Dyon et al, 2021  [11] | 3 (3 ♀; Ø 79) | Interventionsstudie | Dauer der Vokalisationen | 16 Pfund schwere Decke; 10 Minuten Baseline Observierung; danach Proband:innen mit Decke bedeckt; Vokalisationen von außerhalb des Zimmers für 10 Minuten gemessen; danach Decke entfernt; wieder von außerhalb Länge der Vokalisationen gemessen für 10 Minuten; jeder Versuch mit jeder Probandin 6x pro Tag durchgeführt für insgesamt 18 Mal; zehn erfolgreiche Versuche analysiert | Ø Gesamtlänge Vokalisationen Baseline 3.62 Minuten (SD=3.58); Decken 1.8 Minuten (SD=1.85), Abnahme der Decken 0.9 Minuten (SD=0.99);  ↓ mit aufgelegter Decke (p=0.0691); nach Entfernung der Decken Vokalisationen ↓ (p = 0.033) | Mangelnde Akzeptanz der Proband:innen im Umgang mit den Decken; kleine Proband:innengruppe |
| --- | --- | --- | --- | --- | --- | --- |

**Musik und Sound**

| Audiotapes  Burgio et al, 1996  [24] | 13 (12 ♀, 1 ♂; Ø 83.08 Jahre)  (1 Proband:in nur starker Verdacht Demenz aufgrund Symptomatik) | Interventionsstudie | Observierung | Baseline: Verhaltensobservierung für 10 Tage während verschiedener Zeitperioden; Gebirgsbach-/ Ozeantonbänder; während 10-tägigem Versuch max. 4x jeweils beide Audiotapes; jeweils 20 Minuten | Ansprechende (n = 9) und Nicht-Ansprechende (n = 4) bezüglich Intervention  bei n=13 (alle)  Audiotape an:  (M = 51.70%)  Audiotape aus:  (M = 57.61%, p < .001)  ↓ verbaler Agitation  Ozean (p = 0.002) und Gebirgsbach (p =0.06)  Für Ansprechende (n=9):  ↓ verbaler Agitation  Baseline  (M= 50.02%)  Intervention  (M = 38.78%,  p = .001)  ↓11% verbaler Agitation während Analysephase  bei Ansprechrate von 69%,  bezogen nur auf Ansprechende verbale Agitation ↓ 23% | Kleine Proband:innenanzahl; teilweise eingeschränkte Hörqualität; keine Auskunft über Auswirkung auf Lautstärke der Vokalisationen oder Veränderung spezifischer Typen verbaler Agitationen; unverblindet; unterschiedliche Häufigkeit der Versuche pro Proband:in; Audiotapes nur während 51% der Observierungen benutzt; Ablehnung der Kopfhörer |
| --- | --- | --- | --- | --- | --- | --- |
| Gruppenmusik  Lin et al, 2011  [25] | 100 (49 Interventions-gruppe; 51 Kontrollgruppe; 53 ♀; 47 ♂; Ø 82) | Interventionsstudie | Chinesischer CMAI | Interventionsgruppe: 12 Sitzungen (30-minütige Sitzungen 2x Woche für 6 Wochen)  Kontrollgruppe: normale tägliche Aktivitäten | Interventionsgruppe:  ↓verbal nicht-aggressives Verhalten in  6. Sitzung vs. Vortest:  ↓0.22 Punkte (p=0.042)  12. Sitzung vs. Vortest:  ↓0.28 Punkte  (p=0.010)  1 Monat nach Ende  Vs. Vortest:  ↓0.26 Punkte  (p=0.037) | Rekrutierung aus 3 verschiedenen Pflegeeinrichtungen |
| Personalisierte Musik  Isaac et al, 2021  [26] | 74 (51 ♀; 23 ♂; Ø 82.4) | Quasi-experimentelle, nicht-randomisierte Interventionsstudie | Observierung | Personalisierte Musik; 20-30 Minuten Musik hören; 2x/ Tag für acht Wochen  Dazu personalisiertes Behandlungskonzept bei Demenz | verbal agitiertes Verhalten  Baseline:  Repetitive Sätze (60.8%)  Nach 4 Wochen  Repetitive Sätze (27.0%) | Einige Verhaltensweisen wie Schlagen, Treten, Dinge werfen etc. nach vier Wochen zugenommen; keine Kontrollgruppe; mögliche Variationen in Interventions-durchführung; keine Effektauftrennung zwischen Umweltveränderung, Einstellungs-änderung und Musikintervention möglich |

**Massage und sensorische Stimulation**

| Therapeutische Berührung  Woods & Dimond, 2002  [27] | 10 (7 ♀; 3 ♂; Ø 78.9) | Interventionsstudie | Agitated Behavior Rating Scale (ABRS) | sensorische Stimulation durch Hand 5-7 Minuten, 2x/Tag für 3 Tage | Vokalisationen ↓ 48% während 3-tägiger Behandlung; nach Behandlung ↑ | Kleine Proband:innen-gruppe; fehlende Kontrollgruppe; Anstieg von agitiertem Verhalten während 3. Messung nach Versuch |
| --- | --- | --- | --- | --- | --- | --- |
| Multisensorische Stimulation  Maseda et al, 2014  [28] | 30 (27 ♀; 3 ♂; Ø 87.3 Jahre), Follow-up: 26 | Kontrollierte, randomisierte Longitudinalstudie | Spanischer CMAI | Stimulationsgruppe: Sensorische Stimulation z.B. Wassersäulen  2 x/ Woche für 30 Minuten über 16 Wochen  Aktivitätsgruppe: Aktivitäten wie Karten spielen  Kontrollgruppe: normale Tagesroutine | Stimulations- und Aktivitätsgruppe signifikante Effekte für CMAI verbal agitiertes Verhalten  (F2,36 = 10.540, p < .001)  verbal agitiertes Verhalten für Stimulations- und Kontrollgruppe zwischen Woche 0 und Woche 16 ↓  (F2,30 = 5.159,  P= .012)  Keine signifikanten Unterschiede bezüglich CMAI für verbal agitiertes Verhalten nach Intervention (Woche 16) und bei Follow-up ( 8 Wochen später) | Kleine Proband:innenanzahl; scheinbar unterschiedliche Wirksamkeit der Stimulation für kognitive Effekte bei unterschiedlichem Beeinträchtigungs-level (bezieht sich auf hohe kognitive Beeinträchtigung) |
| Multisensorische Stimulation  Sanchez et al, 2016  [29] | 32 (25 ♀; 7 ♂; Ø 85.5), Follow-up 27 | RCT | Spanischer CMAI | Stimulationsgruppe: Sensorische Stimulation z.B. mit Wassersäulen  2x/Woche für 30 Minuten über 16 Wochen  Aktivitätsgruppe: Aktivitäten wie Spiele spielen  Kontrollgruppe: normale Tagesroutine | Stimulations- und Aktivitätsgruppe nicht signifikante Verbesserung von verbal agitiertem Verhalten zwischen Woche 0 und Woche 16  Stimulations- und Kontrollgruppe signifikante Effekte für CMAI verbal agitiertes Verhalten  Zwischen Woche 0 und Woche 16  (F2,36 = 3.460, P= .042, ƞ^2^ =  .155) | Kleine Proband:innen -anzahl; ↑ für Stimulations- und Aktivitätsgruppe für Woche 16 und Follow-up (8 Wochen danach)  (F1,16 = 15.623, P = .042, ƞ^2^ = .456) |

**Öl- und Aromatherapie**

| Essentielle Öle  Leach et al, 2021  [30] | 38 (26 ♀; 12 ♂; Ø 81.71; Kontrollgruppe Ø 82.65) | pragmatische, cluster-randomisierte, placebokontrollierte Pilotstudie | PAS, CMAI | Interventionsgruppe: 1-2 topische Cremes & 1-2 topische Öle;  Kontrollgruppe:  1 Kontrollcreme & 1 Kontrollöl  Beide Gruppen 2.5 ml jeder Creme und jeden Öls 2-4 x täglich;  Interventionsgruppe noch 1-2 ml Salbe; 2-4 x täglich  Insgesamt für 8 Wochen | Signifikanter Gruppeneffekt für PAS aberrante Vokalisationen (p=0.018), ↓ für Interventionsgruppe (Zeit-Gruppen-Interaktion, -0.16; 95% CI -0.03 bis -0.29; p = 0.020) in Woche 4 im Vergleich zur Kontrolle; keine signifikanten Zeit-Gruppen- Interaktionen in Woche 8 und 10  CMAI verbal agitiertes Verhalten statistisch signifikanter Gruppeneffekt (p=0.012) und Zeit-Gruppen Interaktionseffekt (p=0.024)  Interventionsgruppe (Zeit-Gruppen-Interaktion, -5.10; 95% CI -1.44 to -8.76; p = 0.007) in Woche 4 mit Vergleich zur Kontrolle | Möglicherweise Fehler bei Verblindung |
| --- | --- | --- | --- | --- | --- | --- |
| Aromatherapie (Re-view)  Holt et al, 2003  Ballard, 2002  [31] | 72 (60% ♀; Ø 78.4) | RCT | CMAI | 1. 10 % essentielles Melissenöl und Basisöl (200 mg/Tag, zwei Dosen); topisch auf das Gesicht und beide Arme 2x/Tag  2. 10 % Sonnenblumenöl und Basisöl (200 mg/Tag, zwei Dosen), topisch auf das Gesicht und  beide Arme 2x/Tag  über 4 Wochen | CMAI verbal agitiertes nicht-aggressives Verhalten (im Vergleich zur Baseline nach 4 Wochen)  Effekt -2.92 (0.91)  T-Wert = – 3.22  p-Wert = 0.018  95% Konfidenz Limit  -5.14 bis -0.70 | Komedikation zusätzlich zur Aromatherapie; Variationen der verschiedenen Pflege-einrichtungen |

**Psychosoziale Interventionen**

| Schulungsprogramm  Huang et al, 2003  [32] | 48 (30 ♀; 18 ♂; Ø 75.8) | Prospektive, randomi-sierte Interventions-studie | Chinesischer CMAI | Interventionsgruppe:  2 Wochen  Schulungsprogramm für Pflegekräfte + Telefonberatung 1x/Woche für 2 Wochen  Kontrollgruppe:  schriftliche Lehrmaterialien + soziale Telefon-Follow-ups 1x/ Woche für 2 Wochen, keine spezielle Schulung bezüglich Verhaltens-problemen | Interventionsgruppe:  N=24  Verbal agitiertes nicht-aggressives Verhalten  Vortest Durschnitt und (Standardabweichung)  20.33 (8.59)  Erster Nachtest nach 3 Wochen: Durchschnitt und (Standardabweichung) und p-Wert  18.71 (9.07)  p=0.008  Zweiter Nachtest nach drei Monaten: Durchschnitt und (Standardabweichung) und p-Wert  17.50 (8.76)  p=0.010  ↓ zwischen Vortest und erstem Nachtest, ↓ zweiter Nachtest  Kontrollgruppe:  N=24  Verbal agitiertes nicht-aggressives Verhalten  Vortest Durschnitt und (Standardabweichung)  16.38 (8.90)  Erster Nachtest Durchschnitt und (Standardabweichung) und p-Wert  17.21 (8.90)  p= 0.014  Zweiter Nachtest Durchschnitt und (Standardabweichung) und p-Wert  17.63 (9.0)  p=0.067  Keine signifikanten Unterschiede im zweiten Nachtest der Kontrollgruppe | Kleine Proband:innengruppe; relativ kurzes Follow-up der Intervention; Intervention ineffektiv bezüglich physisch-aggressivem Verhalten |
| --- | --- | --- | --- | --- | --- | --- |
| Professionelles Trai-ning  Deudon et al, 2009  [33] | 306 (238 ♀; 68 ♂; Ø 86.5 Interventionsgruppe; Ø 86 Kontrollgruppe) | RCT | CMAI | 90-minütiges professionelles Training für Umgang mit Demenzerkrankten/ BPSD; 2 Stunden/Tag Training 2x/Woche während 1. Monats und dann 1x/Woche während 2. Monats; ∑24 Stunden Training; Follow-up nach 3 Monaten | Zwischen Baseline und Woche 8  ↓Verbal agitiert nicht-aggressives Verhalten  -0.41, (p < 0.001) Für Interventionsgruppe, nicht für Kontrollgruppe (-0.04)  Zwischen Baseline und Woche 20  ↓ -0.47 (p < 0.001)  Nur Interventionsgruppe, nicht Kontrollgruppe (-0.03)  Follow-Up Interventionsgruppe p <0.0001  Kontrollgruppe  p = 0.832 | Verschiedene Baseline Charakteristiken; insgesamt BPSD stärker in Interventionsgruppe; Baseline festgelegt vor Weiterbildung kann zu Problemen bei Randomisierung geführt haben; Proband:innen nehmen trotzdem Psychopharmaka -> keine reine nicht-pharmakologische Studie |

**Tiergestützte Therapie**

| Tiergestützte Therapie  Nordgren & Engström, 2012  [34] | 1 (1 ♀, Ø 84 Jahre) | quasi-experimentelle, longitudinale Fall-Interventionsstudie | Observierung durch Fragebogen | Tiergestützte Therapie mit einem Hund z.B. für Pflege 1x/Woche ca. 60 Minuten für 8 Wochen | Baseline: 1 Woche vor tiergestützter Therapie repetitive Sätze und Fragen mehrmals am Tag; nach tiergestützter Therapie verschwunden und beim Follow-up bei 3 Monaten; jedoch Fluchen beim Follow-up; weniger als 1x pro Woche | nach Therapie Greifen nach Personen; nicht im Follow-up beobachtet; Anstieg von Widerstand beim Be- und Entkleiden direkt nach Intervention; zunehmende Suche nach Aufmerksamkeit; verschiedene unkontrollierbare äußere Einflüsse; Bias durch Bewertungspersonen; nur eine Probandin |
| --- | --- | --- | --- | --- | --- | --- |

**Lichttherapie**

| LED Fototherapie  Chen et al, 2023  [35] | 60 (36 ♀; 24 ♂; Ø 81.23) | RCT | Chinesischer CMAI | Interventionsgruppe: Mit blauem Licht angereicherte Lichttherapie (470 nm maximal, 200 lux) für 60 Minuten/Tag; 10 Wochen für 5 Sitzungen/Woche  Kontrollgruppe: Konventionelles Indoor-Licht von 600 lux mit Farbtemperatur von 3.200K für selbe Dauer | Signifikante Veränderung verbal agitiertes nicht-aggressiven Verhalten direkt nach Beendigung der Intervention  β = −0.63, Waldχ2 = 13.39, p < 0.001  bezogen auf allgemein BPSD keine signifikante Reduktion im 3- und 6 Monate Follow-up | Nebenwirkungen Schwindel (6,7%) und Blendung (10%), Fluktuation neuropsychiatrischer Symptome; visuelle Beeinträchtigung im Alter; kleine Proband:innenzahl; unkontrollierter Wechsel von Medikationsdosierung; unkontrollierte exogene Faktoren wie Bettzeiten etc. |
| --- | --- | --- | --- | --- | --- | --- |

**Elektrokonvulsionstherapie**

| Elektrokonvulsionstherapie (EKT)  Guhra et al, 2018  [8] | 1 (1 ♀, Ø 73) | Fallbericht | Observierung | 4 x EKT mit Thymatron-IV-Gerät, Abstand jeweils drei- fünf Tage | Nach 3x EKT völliges Einstellen des Schreiens; 4 Wochen nach Entlassung keine weiteren Vokalisationen |  |
| --- | --- | --- | --- | --- | --- | --- |
| EKT  Johari et al, 2019  [36] | 1 (1 ♀, Ø 69) | Fallbericht | Observierung | EKT 3x/ Woche für 12 Sitzungen, dann 1xWoche, höhere Energielevel bis zu 160% | vokal-disruptives Verhalten ↓ nach 3x EKT | Insgesamt auf andere Studien bezogen: zusätzlich psychotrope Medikation, inkonsistente Benutzung objektiver Bewertungsskalen, kurzes Follow-up, fehlende Kontrollgruppe, kleine Proband:innengruppe |
| EKT  Ujkaj et al, 2012  [37] | 16 (15 ♀; 1 ♂; Ø 66.6 Jahre) | Retrospektive Datenanalyse | PAS | Durchschnittlich neun EKT-Behandlungen; 12 von 16 Proband:innen bilaterale Behandlung; vier rechts unilaterale Behandlung; danach drei davon bilateral | Aberrante Vokalisationen (repetitive Nachfragen oder Beschwerden, nonverbale Vokalisationen wie Stöhnen oder Schreien)  Vor EKT aberrante Vokalisationen  2.6 (SD ±1.0)  Nach EKT  0.9 (SD ± 0.9)  Abnahme aberranter Vokalisationen  2.6 ±1.0 bis 0.9 ± 0.9 (F = 46.09, df = 1, 15, p <0.001) | Vorrübergehende Verwirrtheit oder schwerwiegendere Verwirrtheit; zunehmende Gangstörung; Fehlende Kontrollgruppe; Konkurrierende Einflüsse wie Alter, Geschlecht oder Pharmakotherapie; kleine Proband:innenen- gruppe; überdurch-schnittlicher Frauenanteil |
